# Integrative network-based approach identifies key genetic elements in breast invasive carcinoma

**DOI:** 10.1186/1471-2164-16-S5-S2

**Published:** 2015-05-26

**Authors:** Mohamed Hamed, Christian Spaniol, Alexander Zapp, Volkhard Helms

**Affiliations:** 1 Center for Bioinformatics, Saarland University, 66041 Saarbrucken, Germany

**Keywords:** Gene Regulatory Network (GRN), epigenetics, miRNA, gene expression, breast cancer, data integration, TCGA, somatic mutations

## Abstract

**Background:**

Breast cancer is a genetically heterogeneous type of cancer that belongs to the most prevalent types with a high mortality rate. Treatment and prognosis of breast cancer would profit largely from a correct classification and identification of genetic key drivers and major determinants driving the tumorigenesis process. In the light of the availability of tumor genomic and epigenomic data from different sources and experiments, new integrative approaches are needed to boost the probability of identifying such genetic key drivers. We present here an integrative network-based approach that is able to associate regulatory network interactions with the development of breast carcinoma by integrating information from gene expression, DNA methylation, miRNA expression, and somatic mutation datasets.

**Results:**

Our results showed strong association between regulatory elements from different data sources in terms of the mutual regulatory influence and genomic proximity. By analyzing different types of regulatory interactions, TF-gene, miRNA-mRNA, and proximity analysis of somatic variants, we identified 106 genes, 68 miRNAs, and 9 mutations that are candidate drivers of oncogenic processes in breast cancer. Moreover, we unraveled regulatory interactions among these key drivers and the other elements in the breast cancer network. Intriguingly, about one third of the identified driver genes are targeted by known anti-cancer drugs and the majority of the identified key miRNAs are implicated in cancerogenesis of multiple organs. Also, the identified driver mutations likely cause damaging effects on protein functions. The constructed gene network and the identified key drivers were compared to well-established network-based methods.

**Conclusion:**

The integrated molecular analysis enabled by the presented network-based approach substantially expands our knowledge base of prospective genomic drivers of genes, miRNAs, and mutations. For a good part of the identified key drivers there exists solid evidence for involvement in the development of breast carcinomas. Our approach also unraveled the complex regulatory interactions comprising the identified key drivers. These genomic drivers could be further investigated in the wet lab as potential candidates for new drug targets. This integrative approach can be applied in a similar fashion to other cancer types, complex diseases, or for studying cellular differentiation processes.

## Background

Breast cancer is one of the most common and predominant cancer types that affects millions of cases and causes thousands of deaths every year [1,2]. With an individual probability of 12% to develop breast cancer, it is the most frequently diagnosed cancer type among women and accounts for the second-highest number of fatalities (15%) of female cancer patients besides lung cancer [3]. Due to its complexity and heterogeneity [4], the molecular mechanism and regulatory patterns underlying breast carcinoma have not been completely unraveled so far.

Treatment and prognosis of cancer development relies largely on a correct classification of the histological grade and identification of the major determinants driving the tumorigenesis process. To better address this, many studies have attempted to build predictive models by analyzing and integrating heterogeneous data sources. For example, Cava et al. presented an effective discrimination of breast cancer types based on a support vector machine classifier combining copy number variations, SNP data, and the expression values of miRNAs, and mRNAs [5]. Also, miRNA-mRNA interactions were combined with transcription factor (TF)-gene interactions to unravel the combinatorial molecular regulations that facilitate progression of colorectal and breast cancer [6,7]. Along the same lines, the integration of gene expression data with protein interaction networks into integrated weighted networks has already proven fruitful in a variety of applications within cancer genomics [8-23]. In general, the combination of microarray studies with mathematical models such as network theory allows to define relationships between genes and to discover interacting networks and pathways, improving the understanding of complex diseases [24].

In recent years, novel network-based approaches have been introduced to improve the understanding of complex human diseases from multiple perspectives. For instance, differential network analysis attempts to better characterize disease phenotypes under two different conditions by studying the changes in the related network interaction patterns [8,9,17,18,25-29]. In cancer genomics, the differential network approach was able to identify essential gene modules that lead to crucial novel biological insights and significant implications for understanding tumorigenesis [9,17,18].

In the light of the recent availability of tumor genomic data and the complexity of the related high throughput analysis, new integrative approaches are needed to boost the probability of successfully identifying the associated genetic key drivers, the causal regulators, the related mutations, biomarkers, and their molecular interactions that potentially drive tumorigenesis. To this end, this study presents an integrative network-based approach based on whole-genome gene expression profiling, DNA methylome, miRNA expression, and genomic mutations of breast cancer samples from the TCGA portal [30]. Based on this, we constructed a gene regulatory network that conforms to the conditions of such biological data and we identified network modules of dysregulated genes. Each module turned out to have distinct functional categories, cellular pathways, as well as oncogene and tumor suppressor specificity. We also extracted breast cancer specific subnetworks from the human genome regulatory interactome induced by the dysregulated miRNAs and the dysregulated mRNAs. Furthermore, we demonstrated a strong association between the different genetic molecules in terms of the interchangeable regulatory effect and genomic proximity. Then, we identified putative genetic key drivers/determinants from genes, miRNAs, and somatic mutations that could possibly drive the oncogenic processes in breast cancer. Our findings are strongly supported by independent evidences. For instance, the protein products of about one third of the identified driver genes are known binding targets of anti-breast cancer drugs, and most of the identified key miRNAs are implicated in cancerogenesis of multiple organs. Moreover, all the identified driver mutations are predicted to cause damaging effects on structures and functions of the related proteins. The rest of the identified driver molecules represent novel potential candidates for new drug targets and further experimental research is warranted to confirm these findings.

## Methods

### Datasets and pre-processing

Data on gene expression, DNA methylation, miRNA expression, and somatic mutations for normal and breast invasive carcinoma samples were collected during May 2014 from The Cancer Genome Atlas (TCGA) [1,30] data portal. All datasets were obtained in level three (log2 transformed and normalized) except the somatic mutations (level two). For consistency, we only considered samples that were common between all four datasets. This yielded in total 151 samples consisting of 131 tumor samples and 20 normal samples (Additional file S1). For both gene expression and methylation datasets, all probes containing NA values or that were annotated to unknown or multiple genes were removed. Also, probes values were merged by computing the mean of all probes related to single genes within a single sample as previously described in [31].

From the DNA methylation data, we kept only those probes representing CpG sites in the promoter regions of genes. For this, we used the transcription start sites (TSS) for all human genes from the Eukaryotic Promoter Database EPD [32]. Promoter regions were defined as an interval of ±2 kb around the TSS as described in [33]. Then we selected only those CpG sites whose genomic coordinates are contained in that interval. The final sizes of the four datasets are listed in Additional file S2.

### Differential analysis

The differential expression/methylation analysis was performed using three methods: 1) Significance Analysis of Microarray (SAM) [34], 2) moderated t-test [35], 3) area under the curve of the receiver operator characteristics (AUC ROC) [35]. Genes that were classified as differentially expressed/methylated genes by at least two of those three methods were included in the list of differentially expressed/methylated genes. The same procedure was applied to determine differentially expressed miRNAs.

### Gene regulatory network construction

The GRN construction involved three steps. First, we constructed the co-expression network from the identified differentially expressed genes based on the topological overlap (TOM) [36] as a distance measure using the WCGNA [37] package in R [38].

In the second step, gene interactions from the co-expression network were connected to regulatory information retrieved from the Transcriptional Regulatory Element Database (TRED) [39], Molecular Signatures Database (MSigDB) [33], and JASPAR database [40]. All genes involved in the co-expression network and listed in at least one of the databases to code for a transcription factor (TF) were marked as TFs. Then, for each TF-gene link in the co-expression network, we searched whether the databases contain a known regulation for this TF-target gene pair. In each of these cases, a directed edge was added between the transcription factor and the target gene. Also, we used the Motif Statistics and Discovery (MoSDi) [41] software to conduct a motif search for all known binding motifs of the TFs represented in the co-expression network against the promoter regions of all genes in the network. If a match was found, a new directed edge from the TF to the gene was added.

In the last step, we constructed a causal probabilistic Bayesian network from the co-expression modules and used the directed edges obtained from step 2 as a start search point to infer directionality between nodes. We used the Sparse candidate [42] algorithm as a search algorithm and the likelihood-equivalence Bayesian Dirichlet (BDe) [43] method as a scoring function for assessing network topology. Also we allowed the following modifiers for a single step in the network search; add edge, remove edge, reverse edge, and swap parent node. For network averaging, we performed the learning approach three times and selected only edges that were inferred at least twice in the three runs (edge confidence level ≥ 66.6%).

As candidate set of directed interactions, we considered directed edges from step 2 as well as directed edges confirmed by both step 1 and step 3. Subsequently, the entire network containing both directed and undirected interactions was exposed to the pruning step explained below. The GRN network was visualized using the igraph [44] package in R.

### Pruning the GRN using methylation and expression profiles

GRN pruning was carried out based on the observation that some genes show increased promoter DNA methylation levels coupled to a remarkable decline of their expression [45]. Based on this, we removed regulatory interactions whose target genes had absolute anti-correlation between their expression and methylation profiles above a selected threshold of 0.65.

### Constructing miRNA-mRNA interactions

The integrated association of the differentially expressed miRNAs and the differentially expressed genes (mRNAs) involved three steps. First, for the set of differentially expressed miRNAs, which were either up- or down-regulated between the tumor and normal samples, we used miRTrail [46] via MicroCosm Targets V5 (http://www.ebi.ac.uk/enright-srv/microcosm/htdocs/targets/v5/) to extract their target mRNAs (regulated genes) and overlapped them with the identified differentially expressed mRNAs. Second, we used the experimentally validated database TransmiR [47] to retrieve the regulatory genes (TFs) that potentially regulate the differentially expressed miRNAs. In both steps, the hypergeometric test with a p-value threshold of 0.05 was applied to test the regulation dependencies between the differentially expressed miRNAs and their target genes/their regulatory TFs. Finally, both miRNA→ mRNA (including TF genes) interaction pairs from step one and TF→ miRNA interaction pairs from step two were joined and merged to a final network.

### Identifying the genetic key drivers/determinants

Key regulators in the constructed networks were identified by determining the minimal set of nodes that regulate the entire network. For this, we used the gplk solver [48] via the numerical optimization package OpenOpt [49].

### Proximity analysis of somatic mutations

The genomic coordinates of the significantly deregulated miRNAs identified in the differential analysis step were downloaded from miRBase [50]. Then, we searched for these sequences in a genomic window of 250 kb around each somatic variant.

To explore possible relationships between differentially methylated CpG sites (identified from the differential analysis step) and somatic mutations, we tested the occurrence of C->A, C->G, and C->T somatic SNVs within a genomic distance of 3 kb from the genomic coordinates of the differentially methylated CpG sites. The selection of the 3 kb genomic distance was based on the maximum considered length of the CpG islands, that is, 500 bp [51] ≤ CpG islands ≤ 3 kb [52]. Ideograms were generated using the circlize [53] package in R. Driver mutations were classified using the CHASM tool [54]. The genomic effect of the driver mutations was analyzed using Ensembl Variant effect Predictor (VeP) [55] that utilizes the functional prediction tools SIFT [56,57] and PolyPhen [58].

### Enrichment and druggability analysis

For gene set enrichment analysis, KEGG pathways and GO functional categories were identified using the DAVID [59] tool as previously described in [60]. Briefly, we determined which pathways/functional terms were annotated to at least two genes and were statistically overrepresented in the study gene set. Enrichment was evaluated through the hyper-geometric test using a p-value threshold of 0.05. For the enrichment analysis of the miRNAs set, we used the TAM [61] online tool. Druggability analysis of the identified driver genes was performed using the PharmGKB [62], CTD [63], and CancerResource [64] databases.

## Results and discussion

### Differential analysis

**Figure 1:**
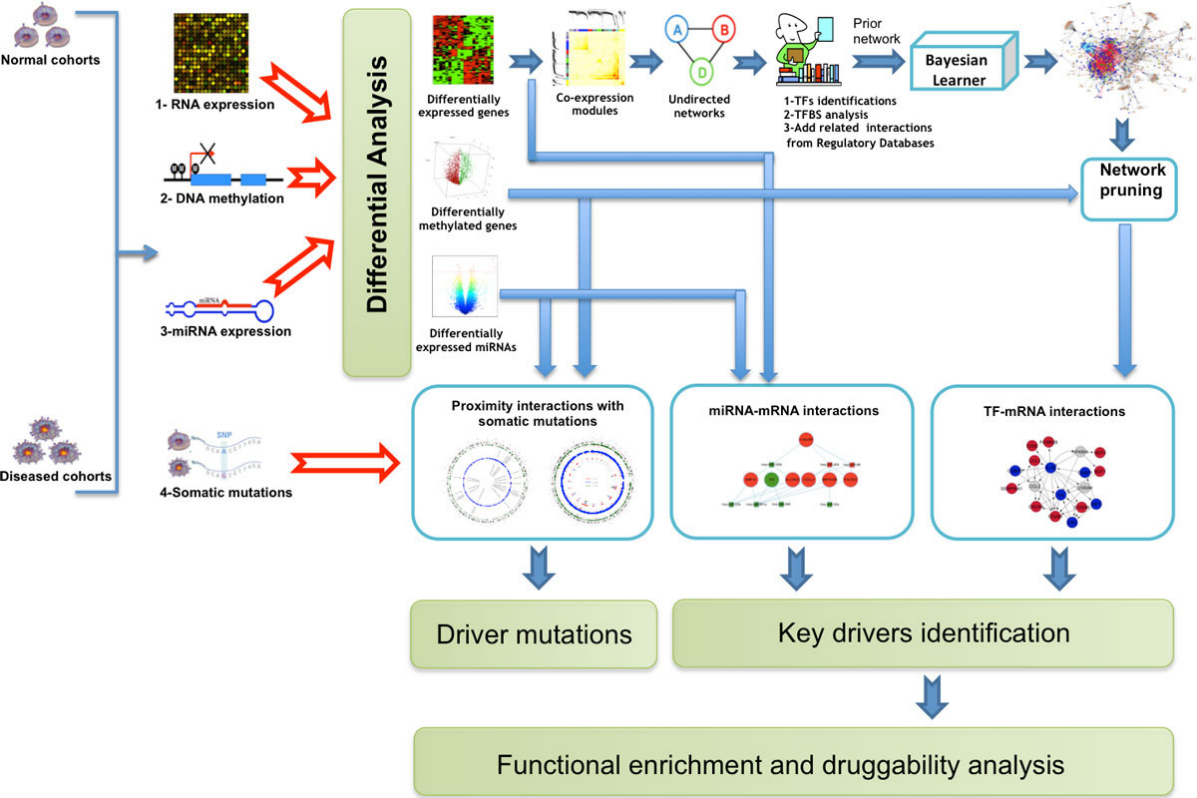
**The integrative network-based approach**. A schematic diagram describing data processing and integration of different data sources to detect major determinants and key driver molecules controlling breast carcinomas.

We developed and applied an integrative network-based approach to conduct combinatorial regulatory network analysis in the context of breast invasive carcinoma with the aim of identifying the major genetic drivers that lead to tumorigenesis (Figure 1). We processed mRNA expression, DNA methylation, miRNA expression, and somatic mutation datasets for 131 tumor samples and 20 control samples of healthy tissues. The differential analysis of the mRNA expression, DNA promoter methylation, and miRNA expression data gave 1317 differentially expressed genes, 2623 differentially methylated genes, and 121 differentially expressed miRNAs, respectively.

### TF-gene interactions

The expression profiles of the 1317 identified differentially expressed genes were used to compute the co-regulation strength between genes using the topological overlap (TOM) measure. Then, we performed hierarchical clustering (HCL) to construct the undirected co-expression network. HCL yielded 10 segregated network modules that contain between 26 and 295 gene members (Table 1). For the seven smallest modules, we collected the related directed regulatory interactions available in three online regulatory databases (JASPAR [40], TRED [39], and MSigDB [33]) and used them as a prior for a Bayesian learner to learn the causal probabilistic regulatory interactions and to generate a directed network topology, (see methods for details). The three largest modules (blue, brown, and turquoise) comprised too many nodes that exceeded the complexity that can be handled by the Bayesian learning approach. Hence, we deliberated the co-expression networks for these three modules by requiring a tighter co-expression threshold and used the obtained network modules for further analysis. It should be mentioned that the Bayesian approach prevents cyclic topology such as self-regulation, which is the case for many genes. Therefore, we note that self-regulatory interactions are not considered in this study. Next, the GRN network modules were pruned in order to maximize consistency between gene expression profiles, methylation fingerprints of gene promoters, and the inferred regulatory interactions. This helps to contextualize the network to the biological experiments from which it was reverse engineered. We removed 89 inferred interactions whose target genes are downregulated and their expression profiles showed absolute anti-correlation measure > 0.65 with their methylation profiles. In those cases we reasoned that downregulation of these target genes was most likely due to their promoter methylation and not due to TF binding [45].

**Table 1:** The key driver elements identified TF-gene interactions and miRNA-mRNA interactions.

	Module	Gene count	Top GO category	Top KEGG categories	Key driver count	Key drivers
**TF-mRNA interactions**	black	41	Regulation of transcription	Pathways in cancer, Renal cell carcinoma	5	SORBS3, ZNF43, ZNF681, RBMX, POU2F1
	
	blue	247	Nucleobase, nucleoside, nucleotide and nucleic acid metabolic process	Cell cycle, Prostate cancer, Melanoma	9	**AR, BRCA1, ESR1, JUN, MYB**, RPN1, E2F1, E2F2, PPARD
	
	brown	195	Anatomical structure morphogenesis	Leukocyte transendothelial migration	5	TMOD3, CREB1, POU5F1, SP3, TERT
	
	green	110	Cellular macromolecule metabolic process	Endometrial cancer, Insulin signaling pathway	15	**B4GALT7, OS9, CDC34**, MAN2C1, MYO1C, SH3GLB2, INPP5E, PLXNB1, USF2, PPP1R12C, CDK9, DAP, E4F1, E2F4, USF1
	
	grey	148	Anatomical structure development	Sulfur metabolism	18	**AHCTF1, NQO2, FGFR2, CCDC130, ABCG4, BIRC6, CA6**, SP4, RNF2, SPRR1B, C16orf65, DNAJC5G, SNCAIP, GRIK5, SLC6A4, SMAD1, DAD1, POU4F2
	
	magenta	26	Regulation of metabolic process	p53 signaling pathway, Alzheimer's disease	3	**ATF6**, NGEF, POGK
	
	pink	30	Transcription initiation from RNA polymerase II promoter	Basal transcription factors	4	**CCDC92**, TMEM70, RNF139, E2F5
	
	red	93	Regulation of cellular process	Endometrial cancer, Neurotrophin signaling pathway	14	**ATP1B1, STAT3, ABCB8, MYC, TGFB1, SP1, TP53**, PCGF1, SUMF2, GTF3A, IPO13, GMPPA, HTR6, TGIF1
	
	turquoise	295	Regulation of cellular metabolic process	p53 signaling pathway, Pancreatic cancer, Apoptosis	2	UBL5, RNF111
	
	yellow	132	Immune system process	Chemokine signaling pathway, Natural killer cell mediated cytotoxicity	19	**APOC1, CD2, CD79B, LRRC28, DAPK1**, FAM124B, EML2, LAP3, TSPAN2, FCRL3, ELMO1, SLC7A7, RASSF5, SLC31A2, TRAF3IP3, GALNT12, ITGA4, SPI1, TFAP2A

	Total	1317				

**miRNA-mRNA interactions**	Genes	**Gene count**	**Top GO category**	**Top KEGG categories**	**Key driver count**	**Key drivers**
		
		869	Regulation of macromolecule metabolic process	Pathways in cancer, Pancreatic cancer, Prostate cancer	17	**MYC, ATG4C, TGFB1, NFKB1, AKT1, EGR1, TP53**, SOX10, SPI1, MECP2, E2F3, CREB1, TCF3, TPP1, FLICE, LPS, PACS1
	
	miRNAs	**miRNA count**	**Top functional categories**	**Top HMDD categories**	**Key driver count**	**Key drivers**
		
		120	miRNA tumor suppressors, immune response, Onco-miRNA, cell death, human embryonic stem cells regulation	Breast cancer (65), Neoplasms (58), Melanoma (56), Ovarian Neoplasms (51), Pancreatic Neoplasms (38), Prostatic Neoplasms (38)	68	mir-126, mir-609, mir-488, mir-191, mir-200c, mir-200a, mir-30a, mir-30d, mir-335, mir-190b, mir-223, mir-106b, mir-519e, mir-210, mir-379, mir-203, mir-205, mir-708, mir-29c, mir-29a, mir-182, mir-183, mir-127, mir-187, mir-425, let-7g, let-7d, mir-152, mir-155, mir-21, mir-22, mir-758, mir-921, mir-922, mir-375, mir-377, mir-181a-2, mir-657, mir-302d, mir-100, mir-10b, mir-10a, mir-625, mir-629, mir-92a-2, mir-26b, mir-25, mir-145, mir-143, mir-141, mir-221, mir-193b, mir-193a, mir-374a, mir-134, mir-146a, mir-31, let-7a-2, mir-27a, mir-27b, mir-133a-1, let-7i, mir-93, mir-23a, mir-148a, mir-196a-2, mir-487b, mir-149

For the 10 gene modules identified in TF-mRNA interactions, we list counts of the involved genes, the most significant GO and KEGG terms, and the identified key driver genes from each module. Similarly for the miRNA-mRNA interactions, we list the key driver molecules of both genes and miRNAs. The driver genes, whose protein products are known to be targeted by drugs, are in bold.

**Figure 2:**
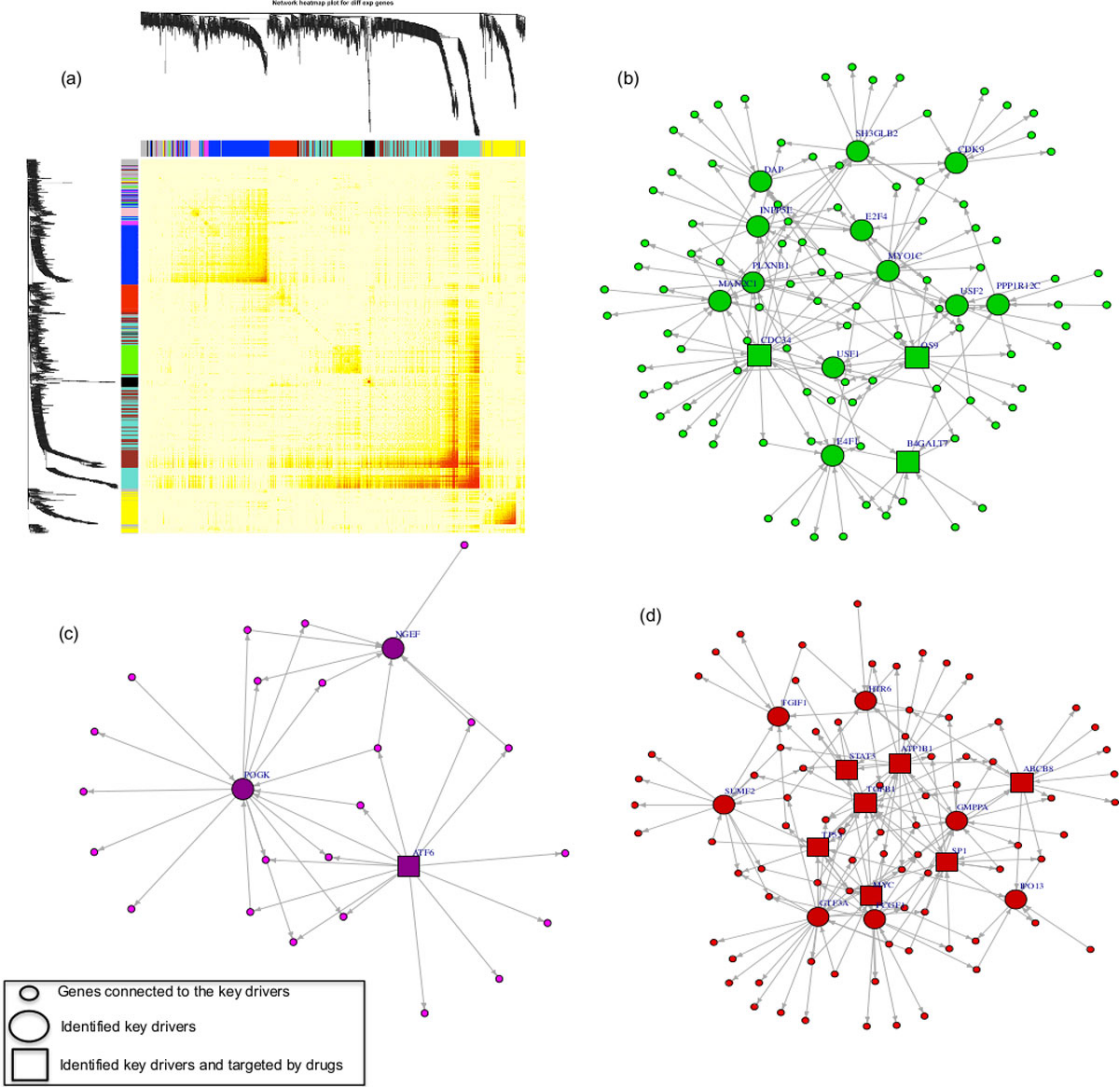
**Gene network modules of TF-gene interactions**. (a) Topological overlap matrix (TOM) heatmap corresponding to the ten co-expression modules. Each row and column of the heatmap represent a single gene. Spots with bright colors denote weak interaction whereas darker colors denote strong interaction. The dendrograms on the upper and left sides show the hierarchical clustering tree of genes. (b), (c), and (d) are the final GRN networks highlighting the identified key drivers genes for the green, magenta, and red modules, respectively. Square nodes denote the identified driver genes that are targeted by drugs. Networks were visualized using the Igraph package in R.

By linking the network modules genes to GO and KEGG annotations via over representation analysis (ORA), we identified the most significant metabolic processes and functional categories that were enriched in each network module and showed relevance to breast cancer, see Table 1. For instance, the red and green modules are enriched with the endometrial cancer pathway, which is tightly associated with breast cancer and subsequent treatment [65]. Also, the magenta and turquoise modules were significantly involved in the p53 signaling pathway, a tumor suppressor gene showing one of the largest frequencies of SNPs among all human genes that have been related to cancer [1]. It has also important roles in diagnosis, in prognostic assessment and, ultimately, in treatment of breast cancer [66-70]. The inferred network topologies for the first three modules (red, green, and magenta) highlighting their identified driver genes are presented in Figure 2. The other network modules are shown in Additional files S3, and S4. Then we utilized the gplk solver [48] via OpenOpt [49] on the 10 inferred network modules to find the minimal set of nodes that dominate and regulate all nodes in each network. In total, we identified 94 key dominating/driver genes in all network modules (Table 1). The follow-up analysis of these driver genes is discussed below.

### miRNA-mRNA interactions

**Figure 3:**
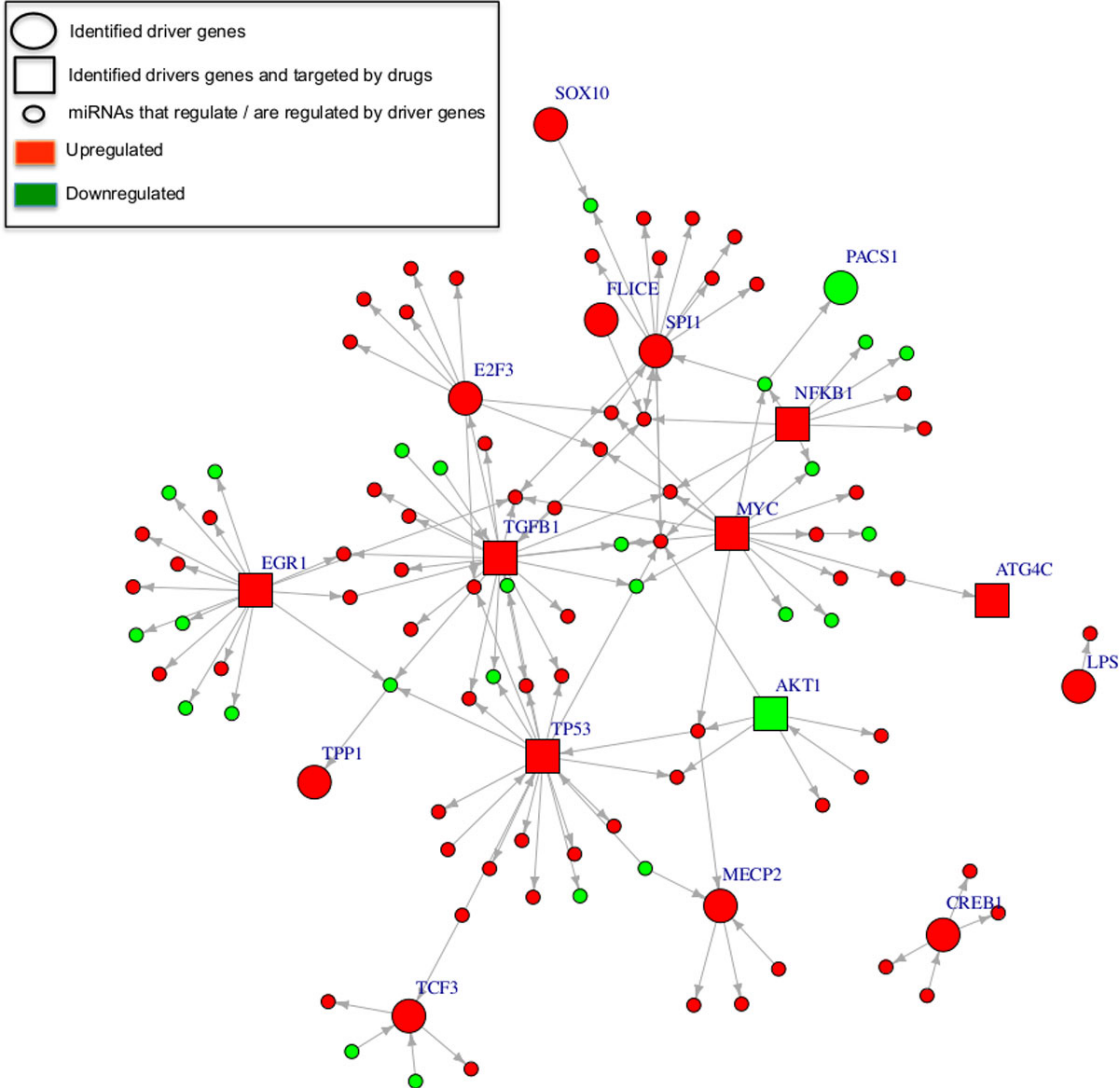
**Regulatory interactions of the 17 key driver genes identified from miRNA-mRNA interactions**. Large nodes represent key driver genes and small nodes represent miRNAs, which regulate or are regulated by these driver genes. Square nodes are the identified driver genes that are targeted by drugs. The network was visualized using the Igraph package in R.

To extract the breast cancer specific subnetworks from the human genome wide regulatory interactome induced by miRNAs and mRNAs, we examined two possible regulation types between the differentially expressed miRNAs and mRNAs: miRNAs regulating target mRNAs and mRNA products (TFs) regulating expression of the miRNAs. We relied on the experimentally validated interactions of both types in building the two networks, (see methods for details). The identified miRNA→mRNA interactions consist of 65 unique miRNAs and 770 unique genes involved in 1949 links. The TF→miRNA interactions include 112 unique TFs and 100 unique miRNAs composing 336 links. A total of 869 genes (including TFs) and 120 miRNAs were present in the combined miRNA→mRNA and TF→miRNA interaction network. 13 mRNAs and 45 miRNAs were common in both interaction types. The 869 genes were mostly involved in regulation of macromolecular metabolic processes and cancer pathways of multiple organs (Table 1). Moreover, the HMDD (Human miRNA Diseases Database) [71] analysis of the 120 miRNAs revealed their implication in cancerogenesis of various organs (Table 1). Next, the two networks comprising the dysregulated miRNAs and mRNAs as well as the interactions among them were combined and further analyzed using OpenOpt [49] and gplk solver [48] to identify genetic drivers and major regulators. This yielded in total 85 key dominating molecules (68 miRNAs and 17 genes) that regulate the entire network nodes (Table 1). The network topologies highlighting the dominating genes and miRNAs are shown in Figure 3 and 3A Additional file S5, respectively.

Interestingly, some of the identified key driver genes such as *MYC, AKT1, and TP53 *were previously implicated and significantly mutated in breast cancer samples [1]. Also the *TCF3 *gene, a well-known TF controlling stem cell identity and self-renewal, is highly expressed in tumor samples and has a central regulatory role in the onset of breast cancer cell differentiation and tumor growth [72]. Additionally, many studies have reported the aberrant expression patterns of the *CREB1 *gene and its role in breast tumor cell growth [73-76] suggesting its protein product as a worthwhile target for anti-cancer drugs [77,78]. It has been demonstrated that the *E2F3 *gene plays a critical role in the transcriptional activation of genes that control the rate of proliferation of tumor cells [79-81]. Furthermore, Vimala et al. [82] recently showed that the *E2F3 *gene is overexpressed in 11 breast cancer cell lines and siRNA-*E2F3 *based gene silencing facilitates the silencing of *E2F3 *overexpression and limits the progression of breast tumors. This strongly conforms to our findings and implies that *E2F3 *may be a potential therapeutic target for human breast cancer. HMDD analysis of the 68 driver miRNAs revealed that 36 miRNAs are involved in breast neoplasms, and the rest are associated with various cancer types such as hepatocellular carcinoma, adenocarcinoma, and prostate cancer. Also the identified key miRNA *mir-29c *as well as the key gene *POU2F1 *have recently been characterized as common hub nodes for three types of breast cancer [7]. Thus, unlike the traditional separate analysis of gene expression profiles [83-87] or the aberration of miRNA expression in cancer tissues [88-90], this integrated molecular analysis of the dysregulated miRNAs and mRNAs was able to uncover important aspects of the miRNA-mRNA interactome, the co-regulation mechanisms, and the underlying pathogenesis of human cancer.

### Proximity analysis of somatic mutations

**Figure 4:**
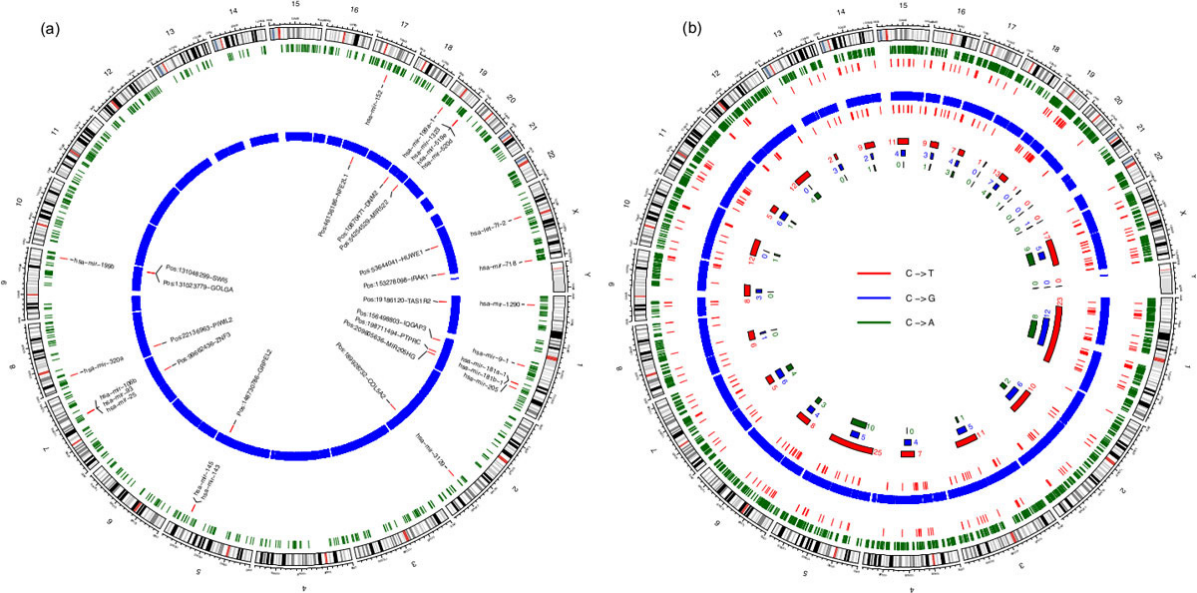
**Proximity analysis of the somatic mutations with the dysregulated miRNAs and differentially methylated genes**. Ideogram plots showing the genomic distribution for (a) the 21 cases of deregulated miRNAs adjacent to somatic mutations. The outer green circle shows the entire dataset of miRNAs, whereas the next highlighted red lines refer to the adjacent deregulated miRNAs (20 miRNAs where one miRNA is matched to 2 SNVs). The inner blue circle represent the entire set of somatic SNVs and the next highlighted red lines depict the SNVs matched to the 21 cases. (b) The 347 cases of somatic mutations occurring in the promoter regions of differentially methylated genes. The outer green circle shows the entire set of differentially methylated genes, whereas the next highlighted red lines refer to the identified cases adjacent to the somatic mutations. The inner blue circle represents the entire set of somatic SNVs and the next highlighted red lines depict the SNVs matched to the identified cases. The plot illustrates also the fractions of the three considered types of mutations (C->T, C->G and C->A) showing the occurrence frequency for each one.

Although next generation sequencing of cancer genomes has unraveled thousands of DNA alterations, the functional relevance of most of these mutations and how they relate to other epigenetic mechanisms (such as DNA methylation and deregulation of miRNAs) are still poorly understood [54]. To this end, we scrutinized whether the significantly differentially expressed miRNAs are in genomic vicinity to the respective somatic variants so that dys-regulation of miRNA expression due to carcinogenesis may depend on the associated nearby somatic variants. We searched for the coding sequences of the dysregulated miRNAs in a genomic window of 250 kb around the somatic variants as previously described in [91]. We detected 21 cases of physical proximity between somatic variants and the deregulated miRNAs (Additional file S6), which are mostly located in chromosomes 1, 7, and 19 (Figure 4-a). These 21 cases encompass 15 distinct mutations and 20 distinct dysregulated miRNAs. To test the significance of these cases, we performed 1000 Wilcoxon tests against random SNV positions considering the same mutation frequency for each chromosome. The deregulated miRNAs identified in the 21 cases were significantly closer to their somatic SNVs pairs in comparison to random SNV positions (p-value equals to 0.001). We also checked whether the non-dysregulated miRNAs (925 miRNAs) are in genomic proximity to the 15 somatic mutations involved in the 21 cases as well. We found that 52 non-dysregulated miRNAs (5.6%) were in vicinity to only 8 mutations so that the other 7 mutations are exclusively associated with the dysregulated miRNAs (Additional file S6).

Similarly, we analyzed the somatic mutations that mainly occur at differentially methylated CpG sites in promoter regions. Overall we identified 347 cases of SNV-differentially methylated gene pairs. These are mostly located on chromosomes 1, 5, and × (Figure 4-b). To address how changes in methylation levels caused by tumorigenesis correlate with mutation rates of different mutation genotypes, we separately analyzed the cases of up- and down-methylated genes. 234 cases involved up-methylated genes, whereas only 113 were associated with down-methylated genes. Generally, mutations in the promoter areas of up-methylated genes occur at a remarkably higher rate than its peers in down-methylated genes especially the C->T genotypes (Additional file S7) since methylated cytosines are prone to thymine transitions by via deamination. This result is in line with the findings of Xia et al. [51] who examined the relationship between DNA methylation and mutation rate. Further, we examined which of the above somatic mutations, which were identified on the basis of their vicinity to either dysregulated miRNAs or differentially methylated genes, could potentially drive tumor cell proliferation in breast cancer. For this, we applied the random forest as a machine learning method implemented in the CHASM tool [54] to distinguish between driver and passenger somatic mutations. As training set, we used the breast cancer labeled data (BRCA) curated from the COSMIC database [92] and provided by CHASM. We identified nine driver mutations (three from miRNA cases and six from differentially methylated gene cases) suggesting their causative role in breast tumorigenesis (Table 2). All these nine mutations are missense and lead to an amino acid substitution. Next, we analyzed the possible impact of the resulting amino acid substitution on structure and function of the respective protein using the PolyPhen [58] and SIFT [56] prediction tools. Interestingly, both methods predict damaging effects of these mutations on protein function conforming their role in driving cancer (Table 2).

**Table 2:** List of the identified driver mutations ordered by CHASM score.

Chrom	Occurring gene	SNV position	CHASM score	P-value	Ref	Alt	Amino acids	Codons	SIFT score	PolyPhen score
1	PTPRC	198711494	0.158	6.00E-04	G	A	E/K	Gag/Aag	Deleterious (0)	probably_damaging (0.999)

8	TNKS	9413850	0.162	6.00E-04	C	T	S/F	tCc/tTc	Deleterious (0.01)	Unknown (0)

X	GRIA3	122319694	0.298	0.0119	C	A	F/L	ttC/ttA	Deleterious (0)	probably_damaging (0.996)

5	PCDHB14	140604126	0.308	0.0134	C	T	S/L	tCg/tTg	Deleterious (0.02)	Benign (0.368)

X	HUWE1	53644041	0.31	0.0136	C	A	R/L	cGa/cTa	Deleterious (0)	probably_damaging (1)

17	NFE2L1	46136186	0.326	0.0175	C	T	S/F	tCc/tTc	Deleterious (0.01)	probably_damaging (0.994)

9	NAIF1	130829249	0.336	0.0204	C	G	K/N	aaG/aaC	Deleterious (0)	probably_damaging (0.995)

2	KLHL23	170592167	0.354	0.0251	C	G	R/G	Cga/Gga	Deleterious (0)	probably_damaging (0.999)

12	KCNA1	5021107	0.384	0.0406	C	T	T/M	aCg/aTg	Deleterious (0)	probably_damaging (0.997)

The CHASM score is defined as the fraction of trees in the Random Forest that voted for the mutation being classified as a passenger. Lower scores increase the confidence of driver mutations. P-values are calculated based on the null score distribution. The table reports also the changes in the related codons and amino acids. The SIFT and PolyPhen scores refer to the prediction of whether an amino acid substitution affects the function and structure of the human proteins. The SIFT prediction is based on the degree of conservation of amino acid residues in sequence alignments derived from closely related sequences (lower scores represent high impacts), whereas the PolyPhen prediction uses physical and evolutionary comparative considerations (higher scores represent high impact and severe influence on the protein function and structure).

### Druggability analysis of protein products of the identified driver genes

As mentioned above, we identified 94 driver genes from the TF-mRNA interactions and 17 driver genes from the miRNA-mRNA interactions. The five well-known breast cancer associated genes *CREB1, MYC, TGFB1, TP53*, and *SPI1 *were common in both sets. Hence, in total 106 driver genes were identified. Also, we characterized 68 dominating miRNAs from the miRNA-mRNA interactions, and nine driver mutations from the proximity analysis. To identify driver genes marked as anti-breast cancer drug-targets, we looked up the drugs and the anti-neoplastic agents that target the proteins corresponding to the 106 driver genes based on the experimentally validated drug-targets reports (see methods). We found that 31% (33 proteins) of the proteins belonging to the identified driver genes are binding targets of at least one anti-breast cancer drug (Additional file S8). These 33 genes are highlighted as square nodes in the network visualizations of TF-mRNA interactions (Figure 2, Additional file S3, and Additional file S4) and miRNA-mRNA interactions (Figure 3). The remaining 73 driver genes were involved in the regulation of biological processes as well as metabolic processes of cancerogenesis in multiple organs such as lung, prostate, and bladder (Additional file S9). This supports the hypothesis that products of the remaining 73 identified driver genes as well as the identified 68 driver miRNAs and the 9 driver mutations may open up new avenues for novel therapeutic drugs.

### Network validation and performance assessment

In order to validate the proposed approach and the constructed network topology [TF-gene interactions only], we applied a peer knowledge-based differential network method, KDDN (Knowledge-Guided Differential Dependency Network) [93] on the same dataset. The same prior was used for KDDN. The networks predicted by our approach showed 61% edges overlap with the inferred differential KDDN interactions due to tumorigenesis.

To assess the reliability of our predictions of key drivers, we further included another differential network method, DiffCoEx (Differential Co-expression Modules) [28] for identifying differential co-expression modules between two biological cohorts. As mentioned above, 33 genes (31%) out of the total 106 driver genes suggested here are known key drivers and are targeted by currently known drugs. In contrast, only 114 KDDN genes (~20%) out of 584 hot spot genes involved in the KDDN network, are binding targets for anti-cancer drugs (Additional file S10). We detected an overlap of 44%, and 16% of the key genes identified by our approach and those obtained by KDDN and DiffCoEx, respectively. DiffCoEx yielded five different modules of genes in which the correlation of gene pairs within the module was significantly different between normal and tumor samples (Additional file S11). Only 151 genes (17%) out of total 886 genes involved in these modules were marked as anti-cancer drug targets. These percentages strongly support the reliability and robustness of our strategy in identifying genomic drivers that could be further experimentally examined as drug targets.

## Conclusions

The enormously increasing availability of transcriptomic and epigenomic data from different biological experiments allow for deep and comprehensive integrative analysis. To this end, this study provides new insights into the complex regulatory mechanisms between gene expression, miRNA biomarkers, epigenetic modifications (represented at the level of DNA methylation) and genetic variants that are associated with the human breast cancer network.

In this work, we demonstrated an integrative network-based approach to conduct combinatorial regulatory network analysis and to identify genomic driver elements that control breast carcinomas. Our results showed a strong association between the regulatory elements of the heterogeneous data sources in terms of the mutual regulatory influence and genomic proximity. By analyzing three different types of interactions, TF-mRNA, miRNA-mRNA, and proximity analysis of somatic variants, we were able to identify various key driver elements (106 genes, 68 miRNAs, and 9 mutations) that could possibly drive breast invasive carcinomas. We also unraveled underlying regulatory interactions among these key drivers and other genetic elements in the breast cancer network. Interestingly, anti-breast cancer drugs target protein products of about one third of the key driver genes and most of the identified key miRNAs are involved in cancerogenesis of multiple organs. Also, the identified driver mutations are predicted to cause damaging effects on protein functions and structures.

These results expand our knowledge base of prospective genomic drivers and provide encouraging support that many of the novel identified genetic elements are potential targets for new drugs. We note that these key drivers were identified based on the presented computational framework and further wet lab work is warranted to confirm their efficacy as putative anti-cancer drug targets. Especially when combined with experimental validation, this network-based approach could promote novel insights on cancer genomic data to develop new therapeutic strategies and thus better treatment. Finally, this approach can be applied to other cancer types or complex diseases and could be extended for studying cellular development as well.

## List of abbreviation

**TCGA**: The Cancer Genome Atlas

**TF**: Transcription factor

**GRN**: Gene regulatory network

**GO**: Gene Ontology

**KEGG**: Kyoto Encyclopedia of Genes and Genomes

**TOM**: Topological overlap matrix

**HMDD**: Human MicroRNA Disease Database

**KDDN**: Knowledge-Guided Differential Dependency Network

**DIffCoEx**: Differential Co-expression Modules

**Ensembl VeP**: Ensembl Variant effect Predictor

## Competing interests

The authors declare that they have no competing interests.

## Financial disclosure

None

## Authors' contributions

MH designed the study and developed the main pipeline used in the analysis. CS participated in identification of key drivers and writing the manuscript. AZ carried out the proximity analysis. VH revised and helped to draft the manuscript. All authors read and approved the final manuscript.

## Supplementary Material

Additional file S1 **TCGA barcodes of the samples considered in our analysis**. We selected only normal and tumor samples from the TCGA portal where all four datasets have been measured. Click here for file

Additional file S2 **Size of datasets after the pre-processing step**. Click here for file

Additional file S3 **The inferred regulatory networks for the black, pink, grey, and yellow gene modules**. For clarity, we visualized only the identified key driver genes and the nodes connected to them. Click here for file

Additional file S4 **The co-expression networks of the brown, turquoise, and blue gene modules**. Due to the large sizes of those modules, the Bayesian approach was not able to infer causal interactions among them. Therefore we display the co-expression networks for these three modules. For clarity, we visualized only the identified key driver genes and the nodes connected to them. Click here for file

Additional file S5 **Regulatory interactions of the identified 68 key miRNAs from the miRNA-mRNA interactions**. Large nodes represent the 68 key miRNAs and smaller nodes represent the TFs or mRNAs that regulate or are regulated by these key miRNAs. Click here for file

Additional file S6 **The deregulated miRNAs in proximity to somatic mutations**. 21 cases of miRNA-SNV pairs were identified. The genomic distance between miRNAs and SNVs is reported in base pairs. SNVs marked with (*) are the exclusive ones associated only with the dysregulated miRNAs and not with any of the non-dysregulated miRNAs. Click here for file

Additional file S7 **Proximity analysis of somatic mutations with the up-and down-methylated genes**. Ideogram plots showing the genomic distributions of the somatic mutations occurring at promoter regions of (a) the up-methylated genes (234 cases), and (b) down-methylated genes (113 cases). The outer green circle shows the entire set of differentially methylated genes, whereas the next highlighted red lines refer to the identified cases adjacent to the somatic mutations. The inner blue circle represents the entire set of somatic SNVs and the next highlighted red lines depict the matched SNVs in the identified cases. The plot illustrates also the fractions of the three considered types of mutations (C->T, C->G and C->A) showing the occurrence frequency for each. Obviously the C->T mutations for the up-methylated genes occur at a higher rate than its peers in the down-methylated genes. Click here for file

Additional file S8 **A list of the 33 genes whose gene products are targeted by anti-cancer drugs, characterized from the three considered drug databases, CTD, PharmGKB, and Cancer resource**. (1) means that at least one drug that targets this gene product is reported in this database, and (0) means no drugs are reported for the respective gene in this database. Not included are substances that are known to be cancerogenous or mutagenic. Click here for file

Additional file S9 **Ten most significant GO terms and KEGG pathways enriched in the list of the 73 candidate driver genes**. Click here for file

Additional file S10 **The network inferred using the KDDN method**. For clarity, we visualized only the known drug target genes (red and labelled) and the genes connected to them (green). Click here for file

Additional file S11 **The network modules inferred using the DiffCoEx method**. Each network corresponds to the highlighted module color in the heatmap. For clarity, we visualized only the known drug target genes (labelled and square nodes) and the genes connected to them. Click here for file

## Declarations

Publication costs for this article were funded by Saarland University, Saarbrucken, Germany.

This article has been published as part of *BMC Genomics *Volume 16 Supplement 5, 2015: Proceedings of the 10th International Conference of the Brazilian Association for Bioinformatics and Computational Biology (X-Meeting 2014). The full contents of the supplement are available online at http://www.biomedcentral.com/bmcgenomics/supplements/16/S5.
