# Supplementary material for: Integrative network-based approach identifies key genetic elements in breast invasive carcinoma
Source: BMC Genomics. 2015 May 26;16(Suppl 5):S2. doi: 10.1186/1471-2164-16-S5-S2 (PMC4460623; doi:10.1186/1471-2164-16-S5-S2)
Supplement: Additional file S2 — Size of datasets after the pre-processing step. [file 1471-2164-16-S5-S2-S2.pdf]

**Data set**	**Dataset Size**	**Platform**
Gene expression	17570 genes * 151 samples	AgilentG4502A_07_3
DNA methylation	8250 genes * 151 samples	Illumina_Infinium_DNA methylation array (HumanMethylation27, HM27)
miRNA expression	1046 miRNAs * 151 samples	IlluminaHiSeq_miRNASeq
Somatic mutation	90489 somatic mutations between the tumor and normal samples	IlluminaGA_DNASeq_curated
