# Supplementary figures and images for: Integrative network-based approach identifies key genetic elements in breast invasive carcinoma
Source: BMC Genomics. 2015 May 26;16(Suppl 5):S2. doi: 10.1186/1471-2164-16-S5-S2 (PMC4460623; doi:10.1186/1471-2164-16-S5-S2)

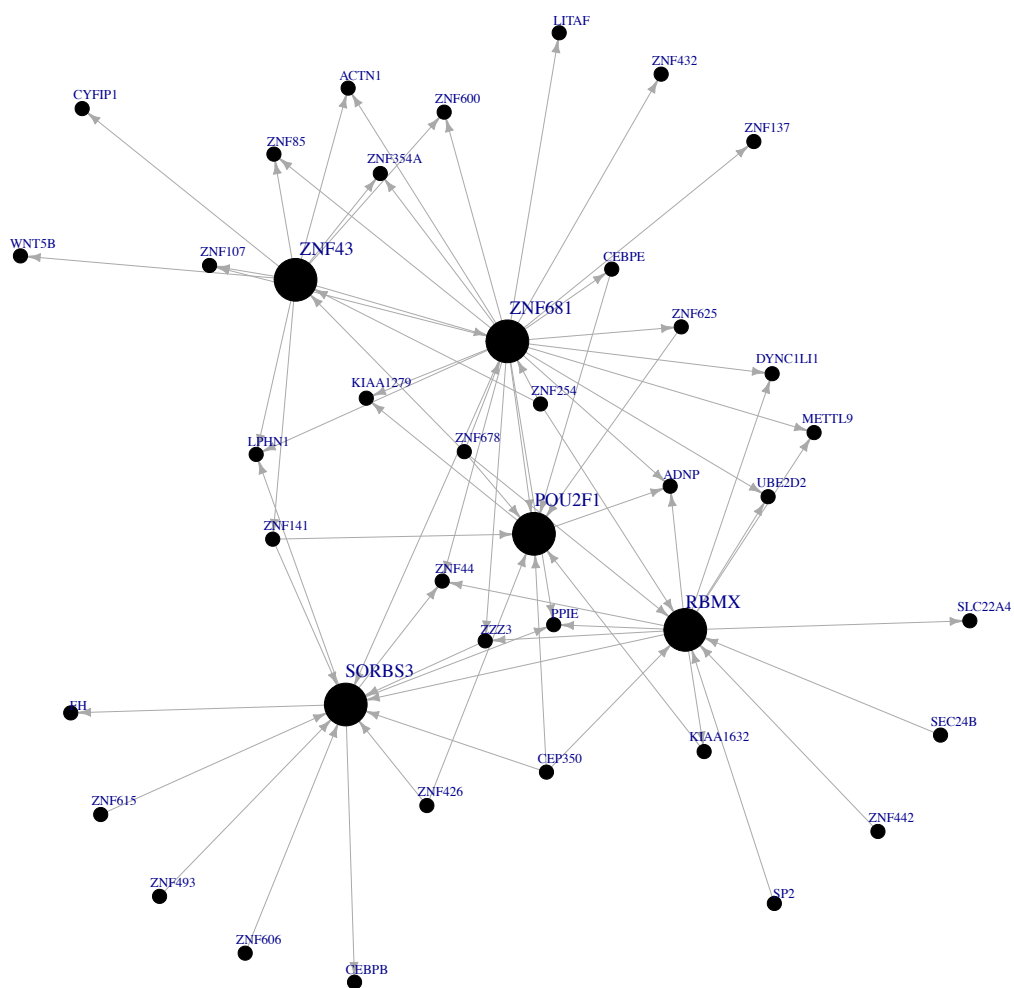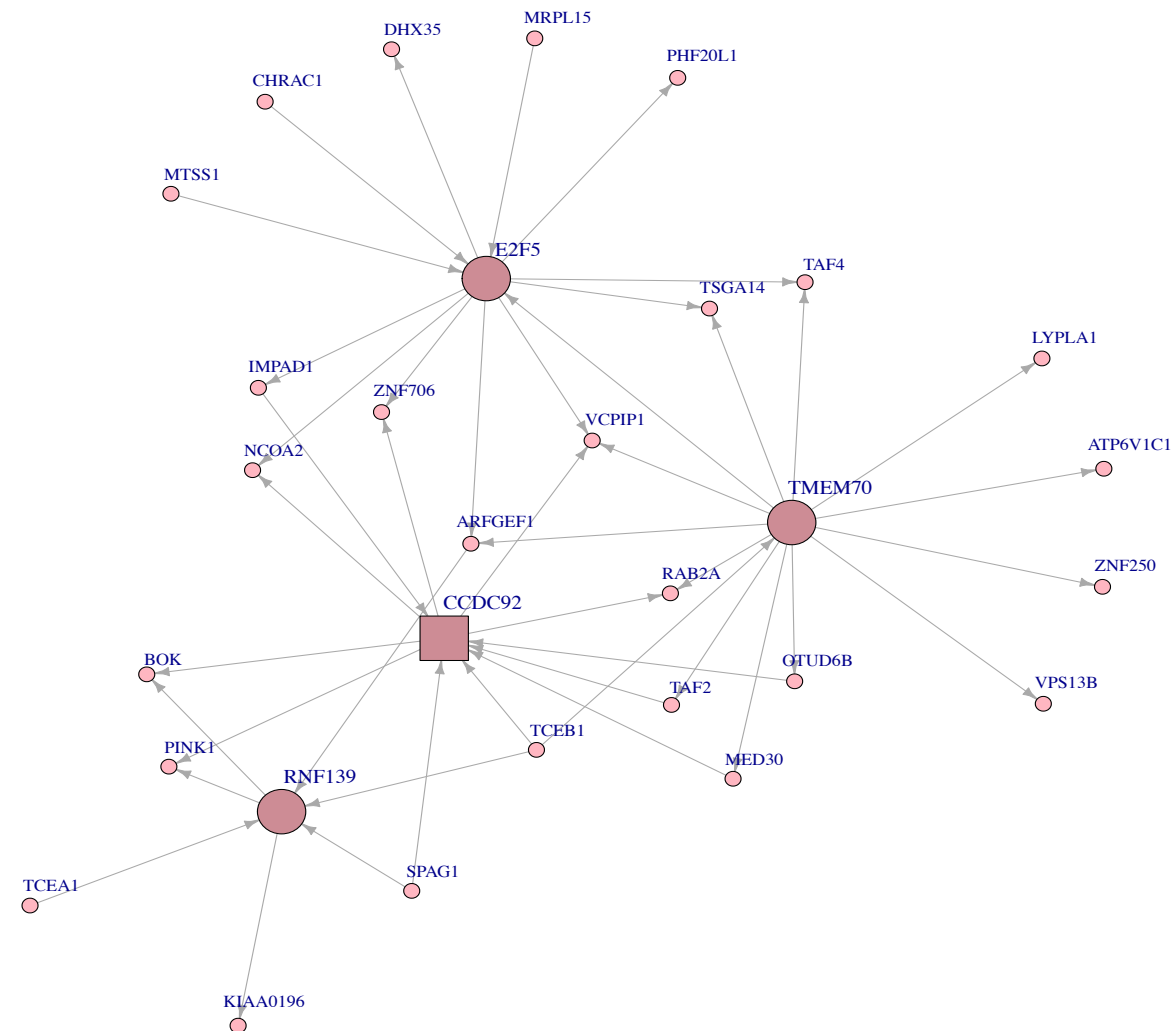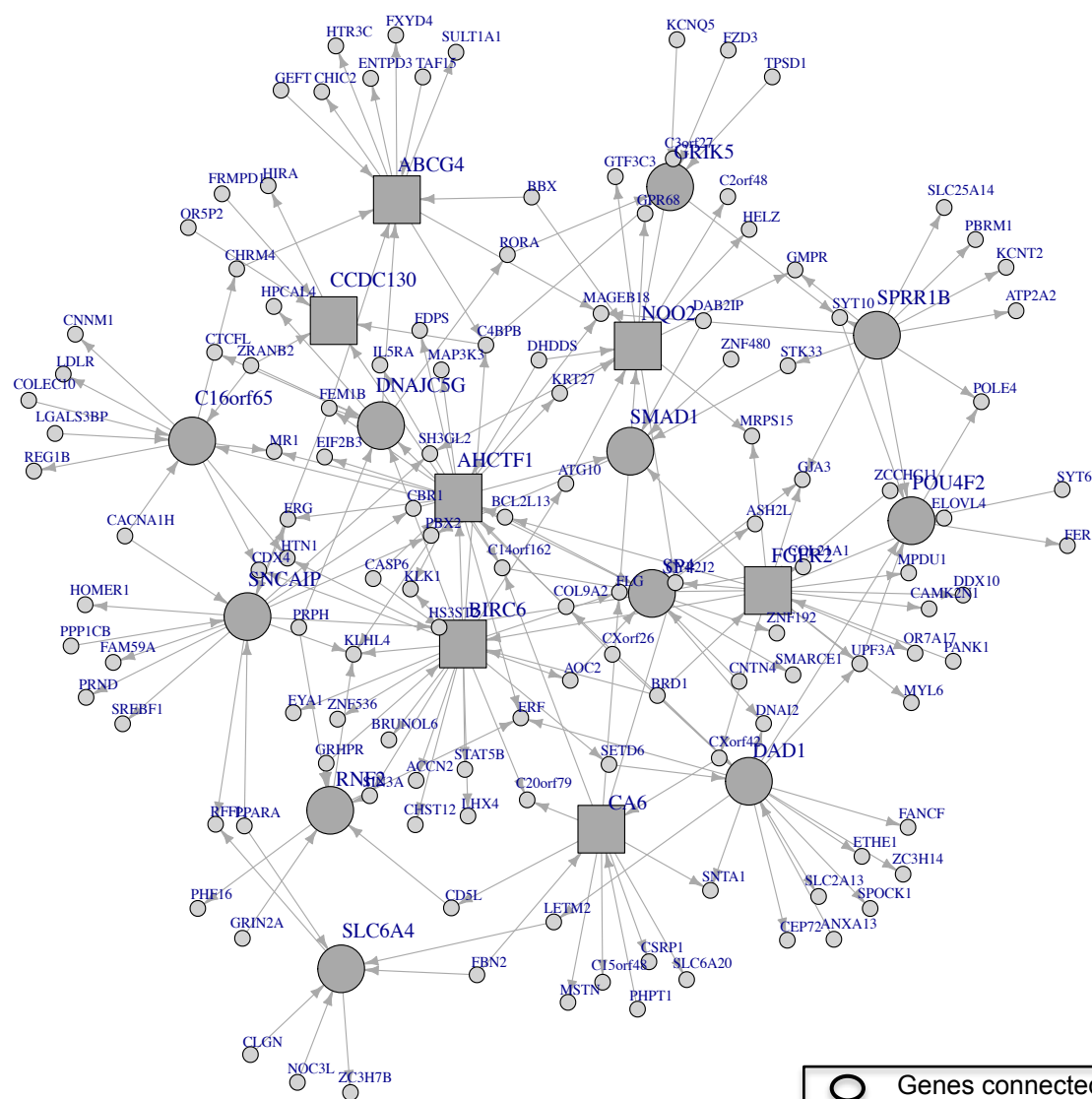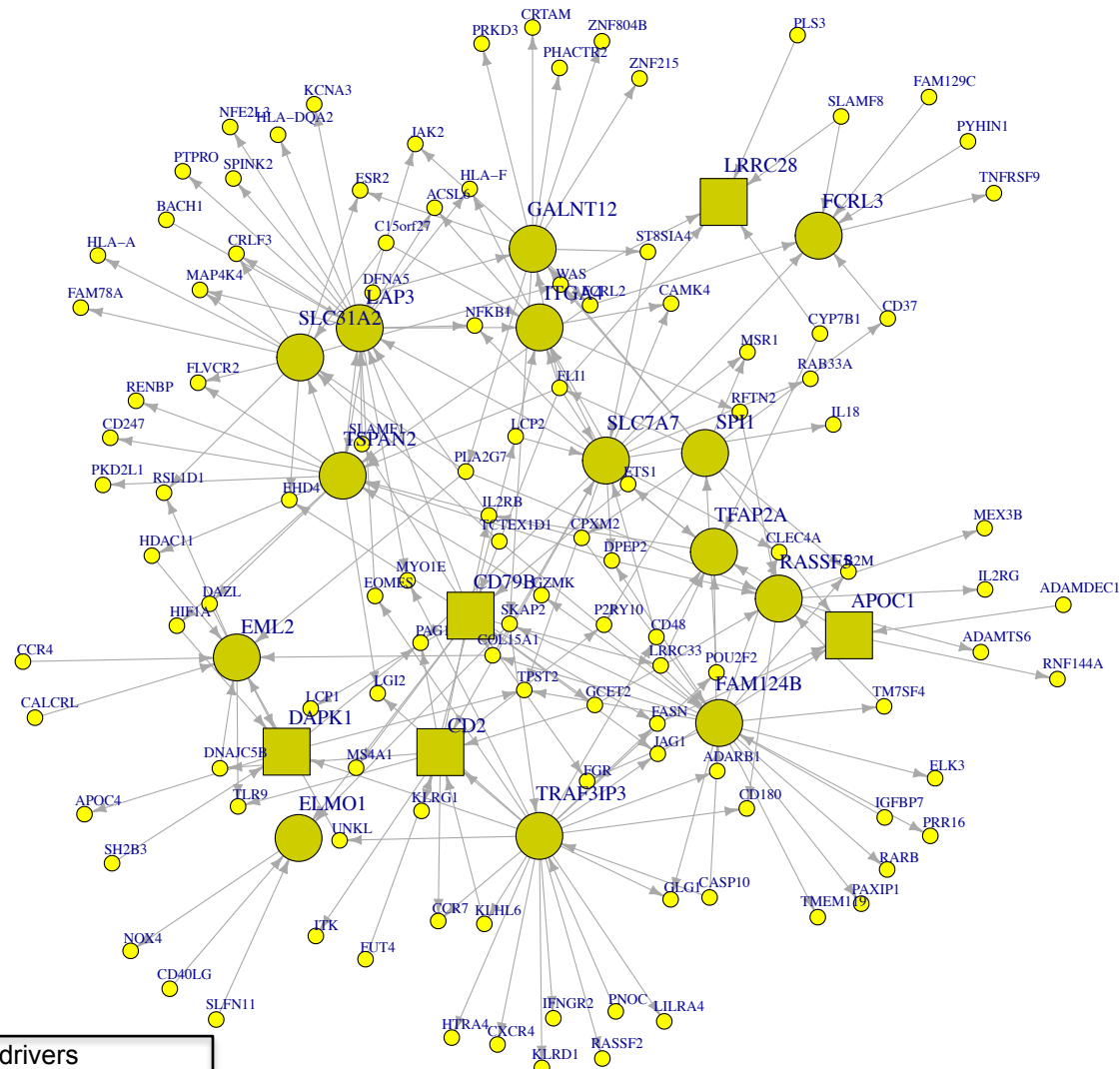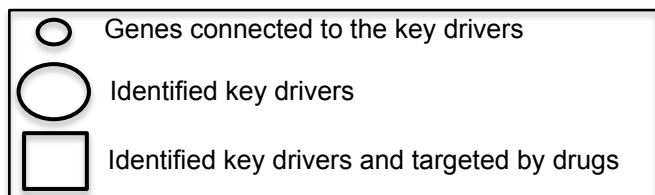

Supplement: Additional file S3 — The inferred regulatory networks for the black, pink, grey, and yellow gene modules. For clarity, we visualized only the identified key driver genes and the nodes connected to them. [file 1471-2164-16-S5-S2-S3.pdf]

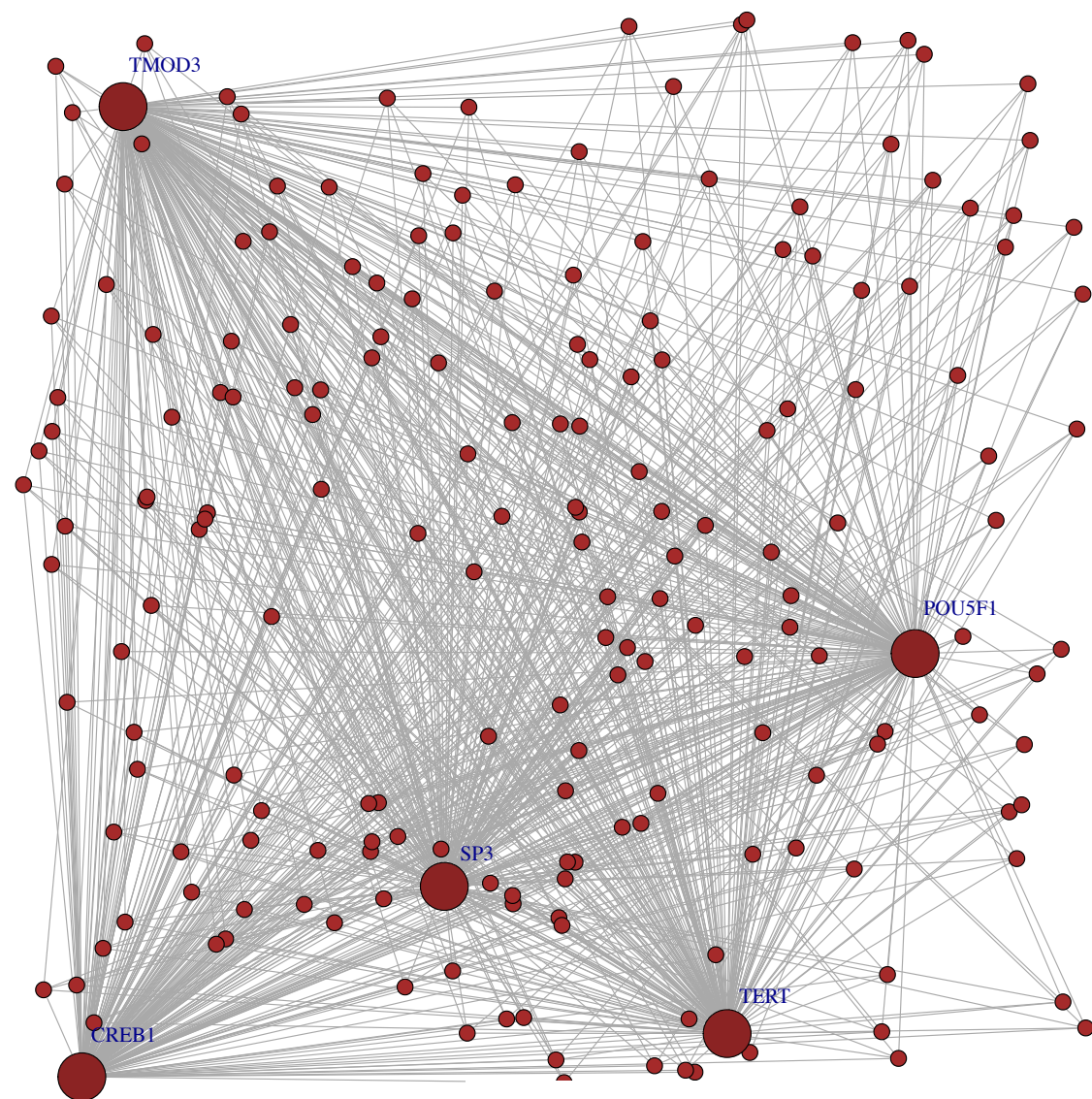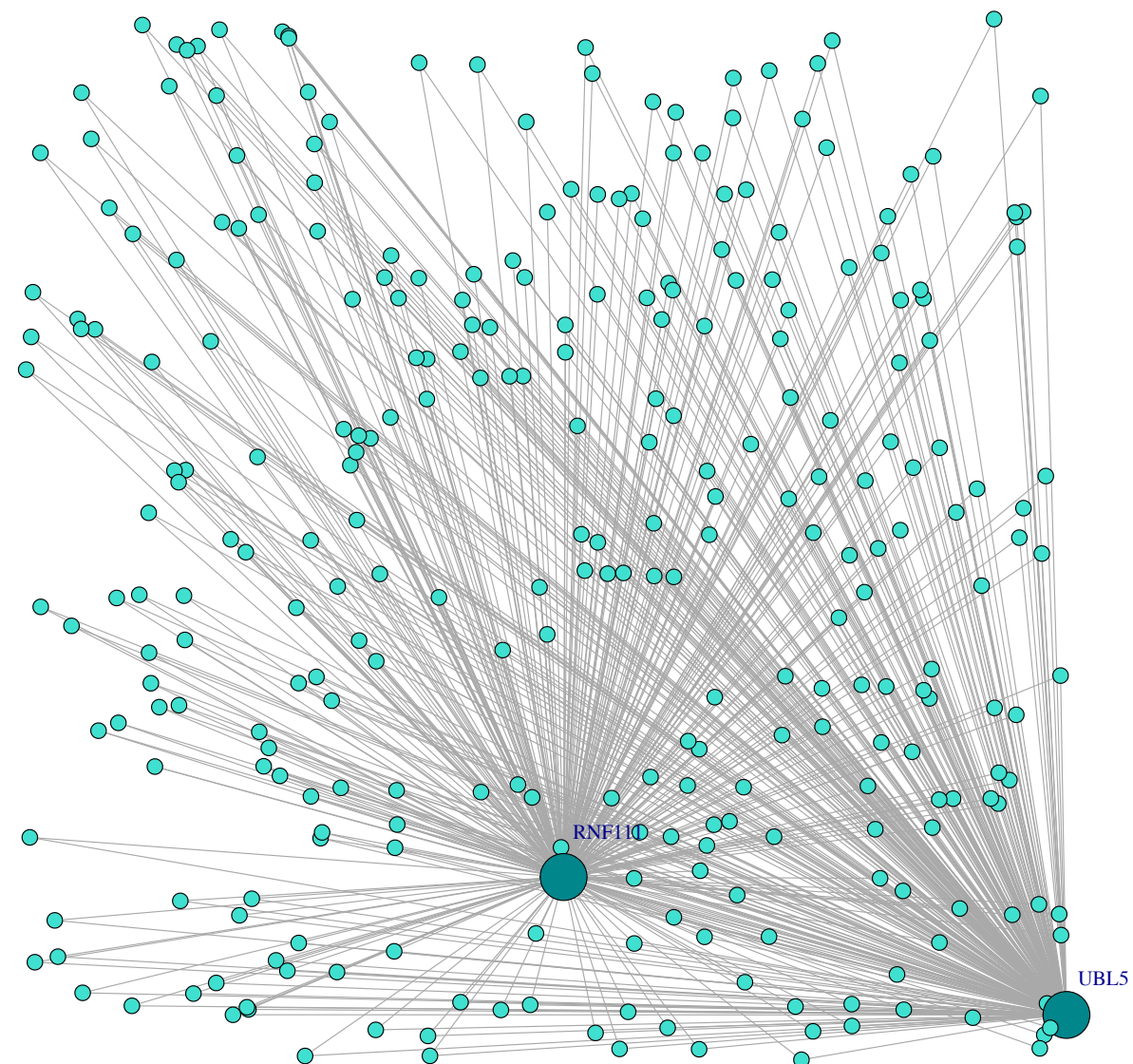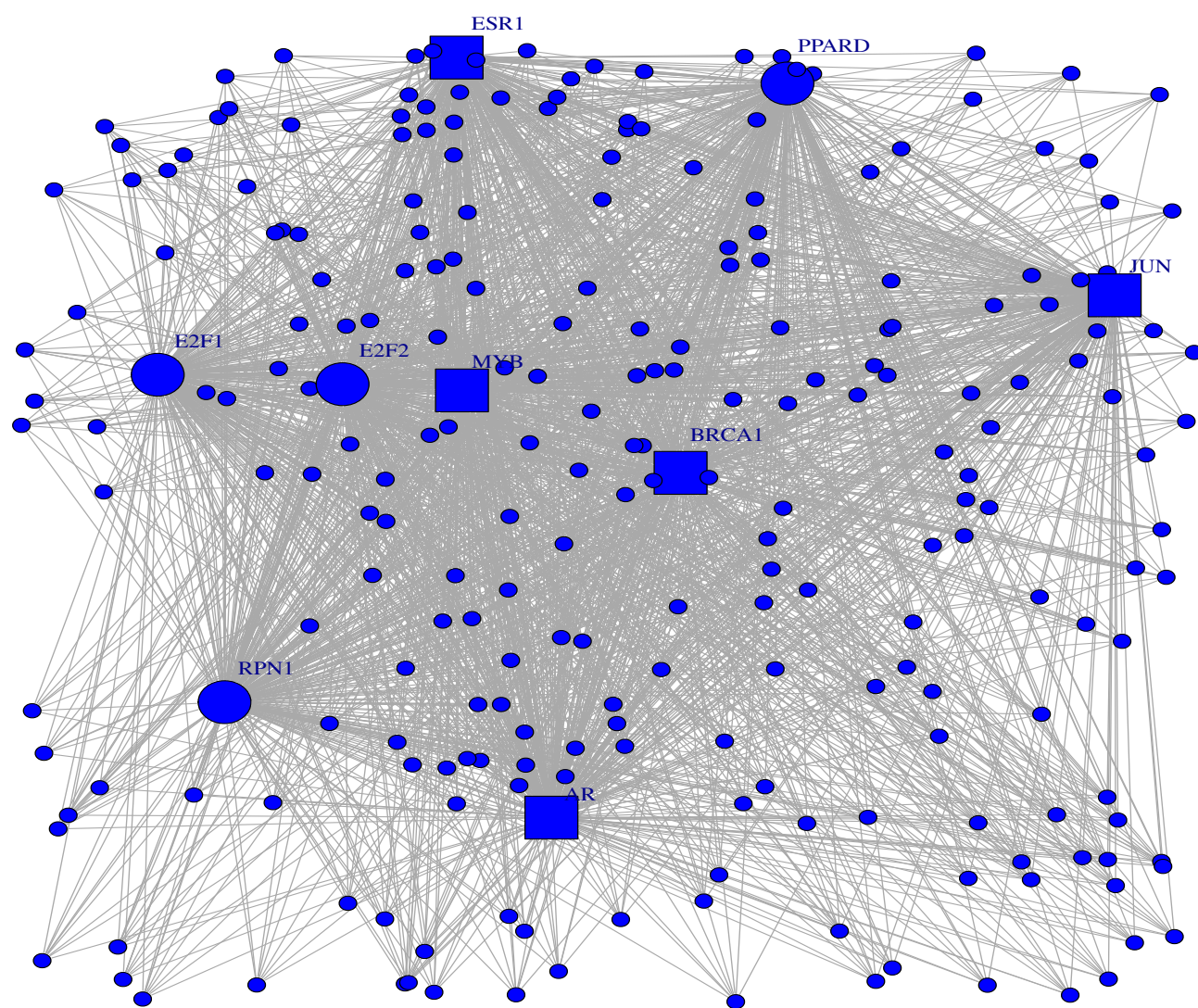

Supplement: Additional file S4 — The co-expression networks of the brown, turquoise, and blue gene modules. Due to the large sizes of those modules, the Bayesian approach was not able to infer causal interactions among them. Therefore we display the co-expression networks for these three modules. For clarity, we visualized only the identified key driver genes and the nodes connected to them. [file 1471-2164-16-S5-S2-S4.pdf]

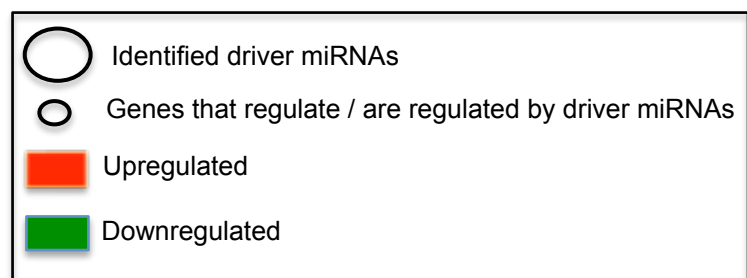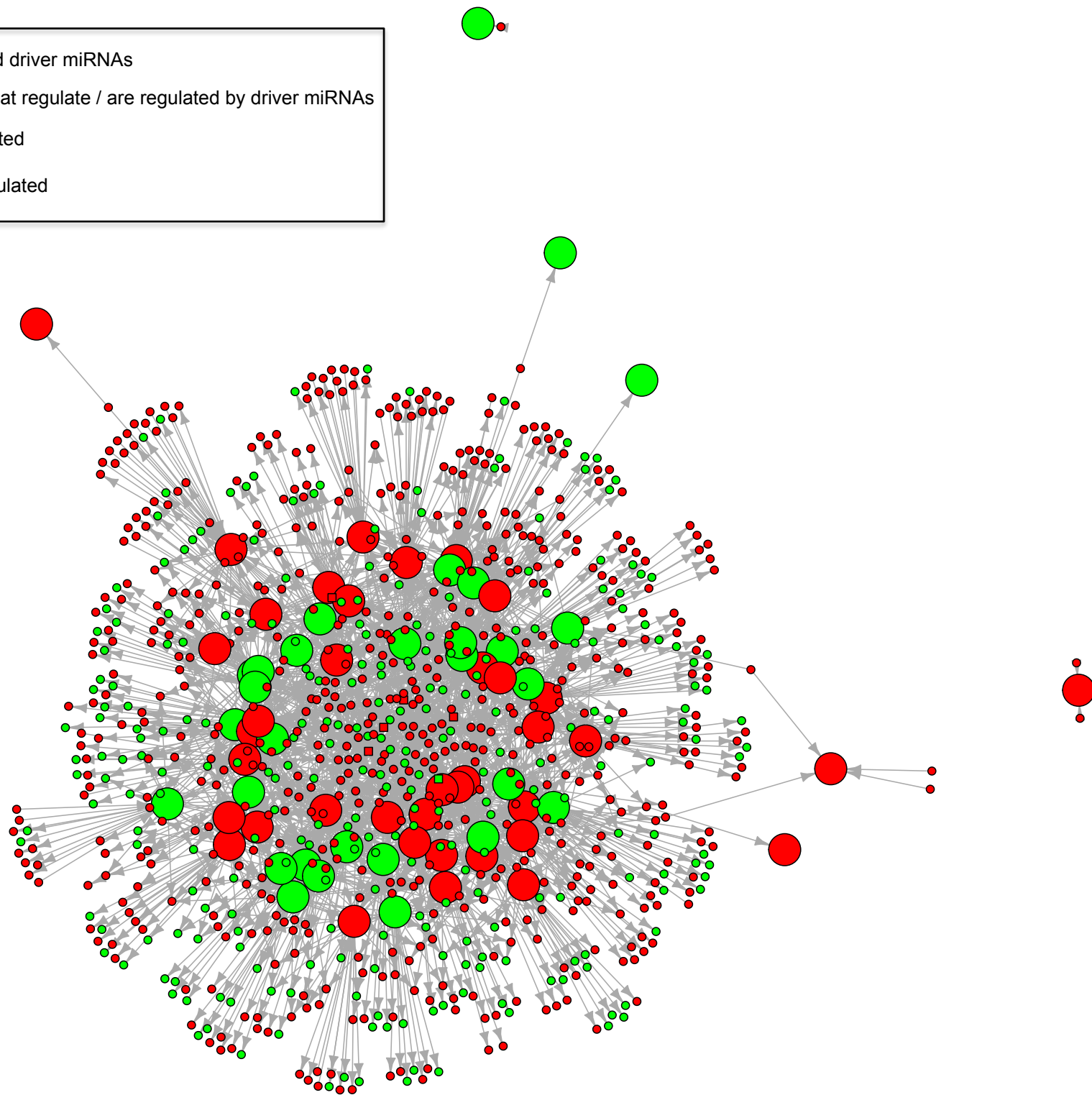

Supplement: Additional file S5 — Regulatory interactions of the identified 68 key miRNAs from the miRNA-mRNA interactions. Large nodes represent the 68 key miRNAs and smaller nodes represent the TFs or mRNAs that regulate or are regulated by these key miRNAs. [file 1471-2164-16-S5-S2-S5.pdf]

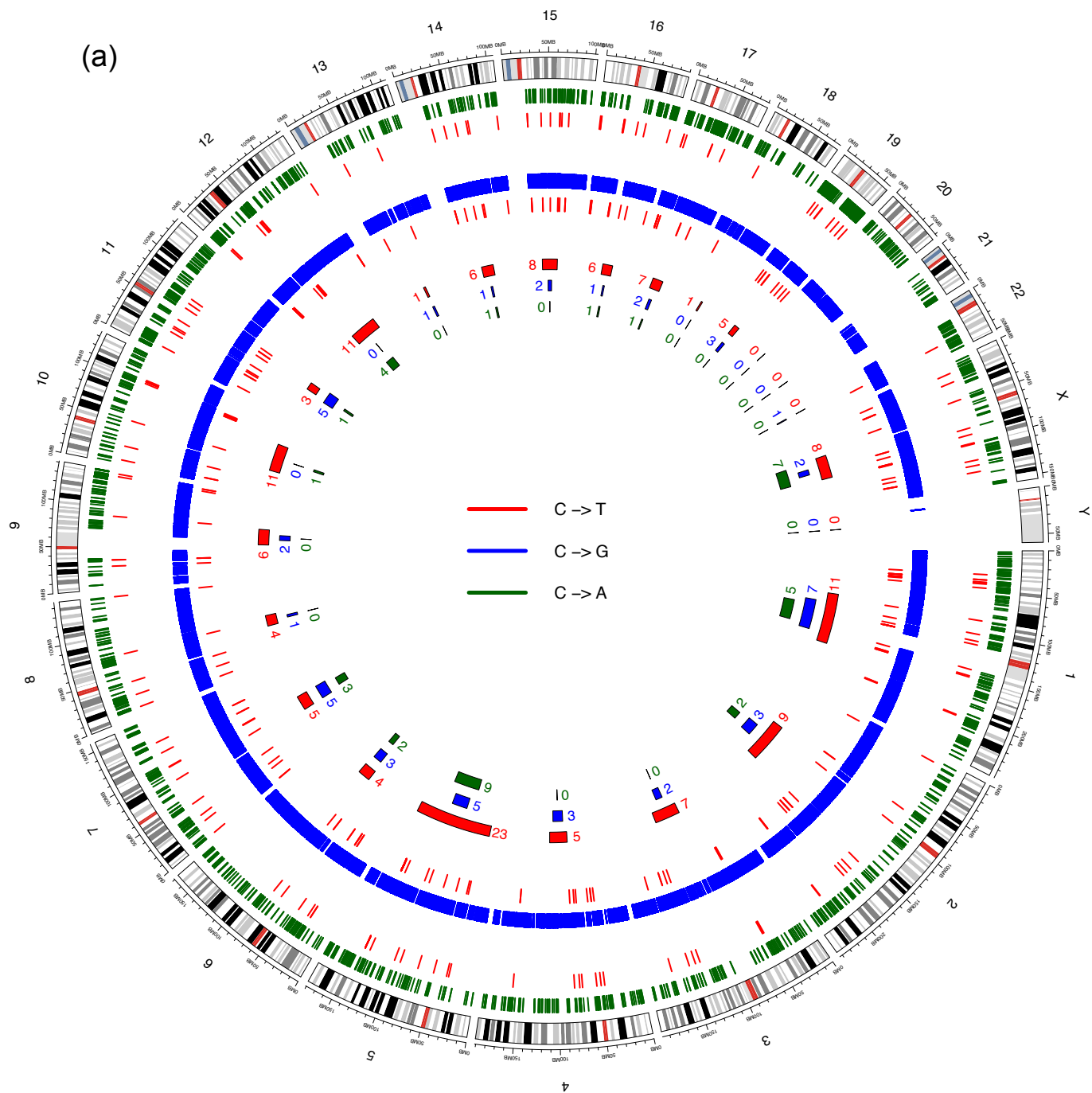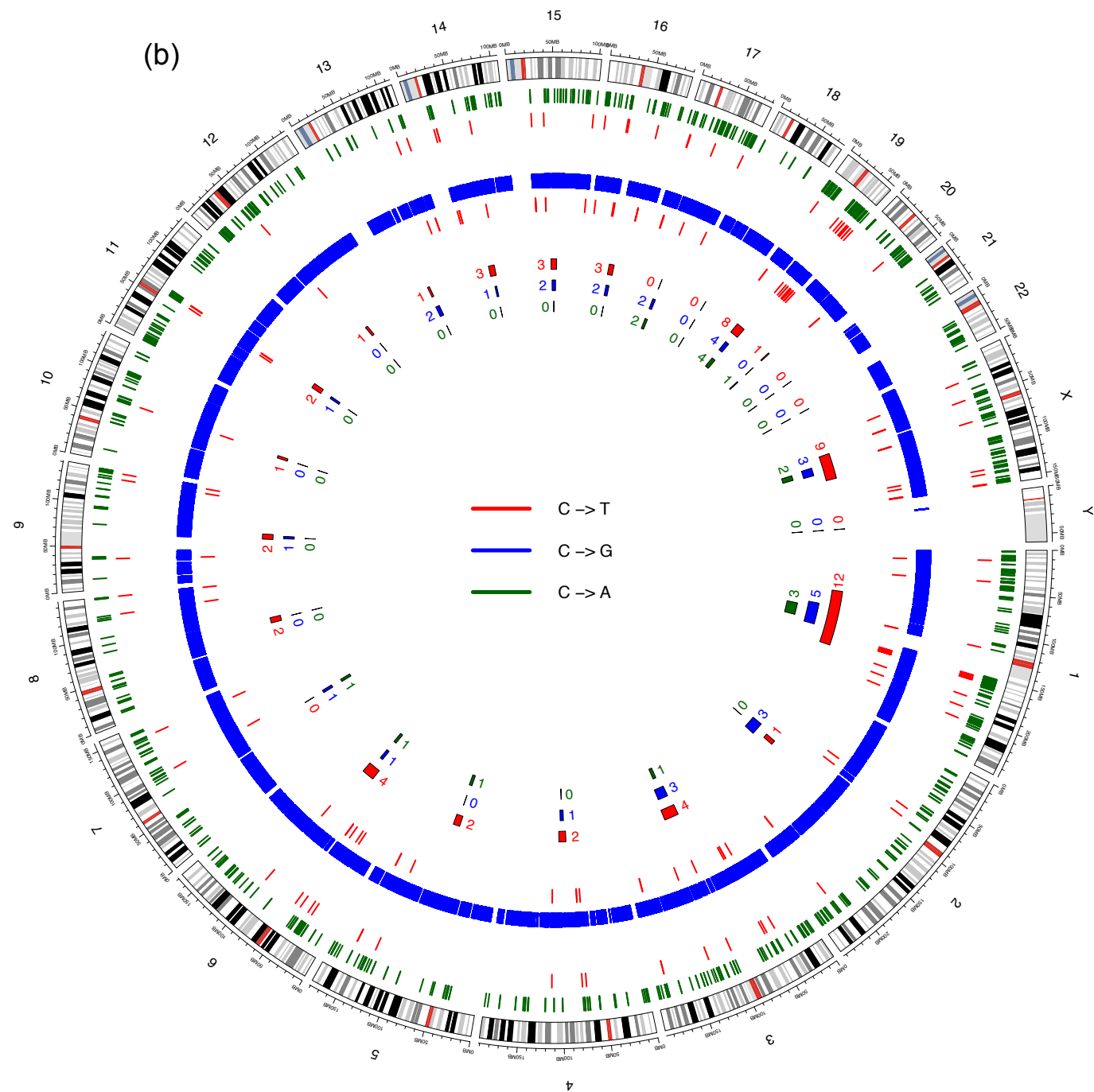

Supplement: Additional file S7 — Proximity analysis of somatic mutations with the up-and down-methylated genes. Ideogram plots showing the genomic distributions of the somatic mutations occurring at promoter regions of (a) the up-methylated genes (234 cases), and (b) down-methylated genes (113 cases). The outer green circle shows the entire set of differentially methylated genes, whereas the next highlighted red lines refer to the identified cases adjacent to the somatic mutations. The inner blue circle represents the entire set of somatic SNVs and the next highlighted red lines depict the matched SNVs in the identified cases. The plot illustrates also the fractions of the three considered types of mutations (C->T, C->G and C->A) showing the occurrence frequency for each. Obviously the C->T mutations for the up-methylated genes occur at a higher rate than its peers in the down-methylated genes. [file 1471-2164-16-S5-S2-S7.pdf]

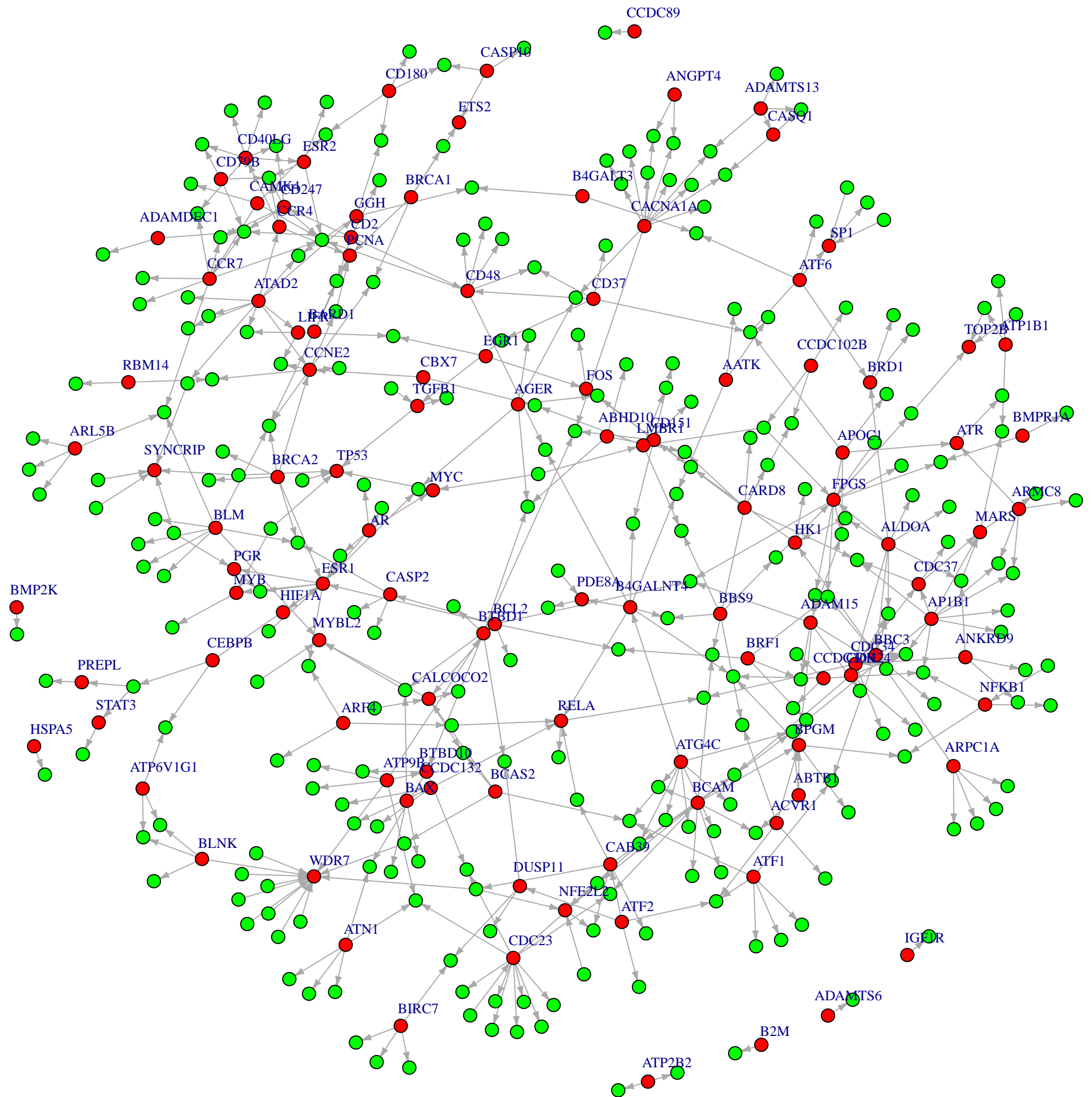

Supplement: Additional file S10 — The network inferred using the KDDN method. For clarity, we visualized only the known drug target genes (red and labelled) and the genes connected to them (green). [file 1471-2164-16-S5-S2-S10.pdf]

Normal samples

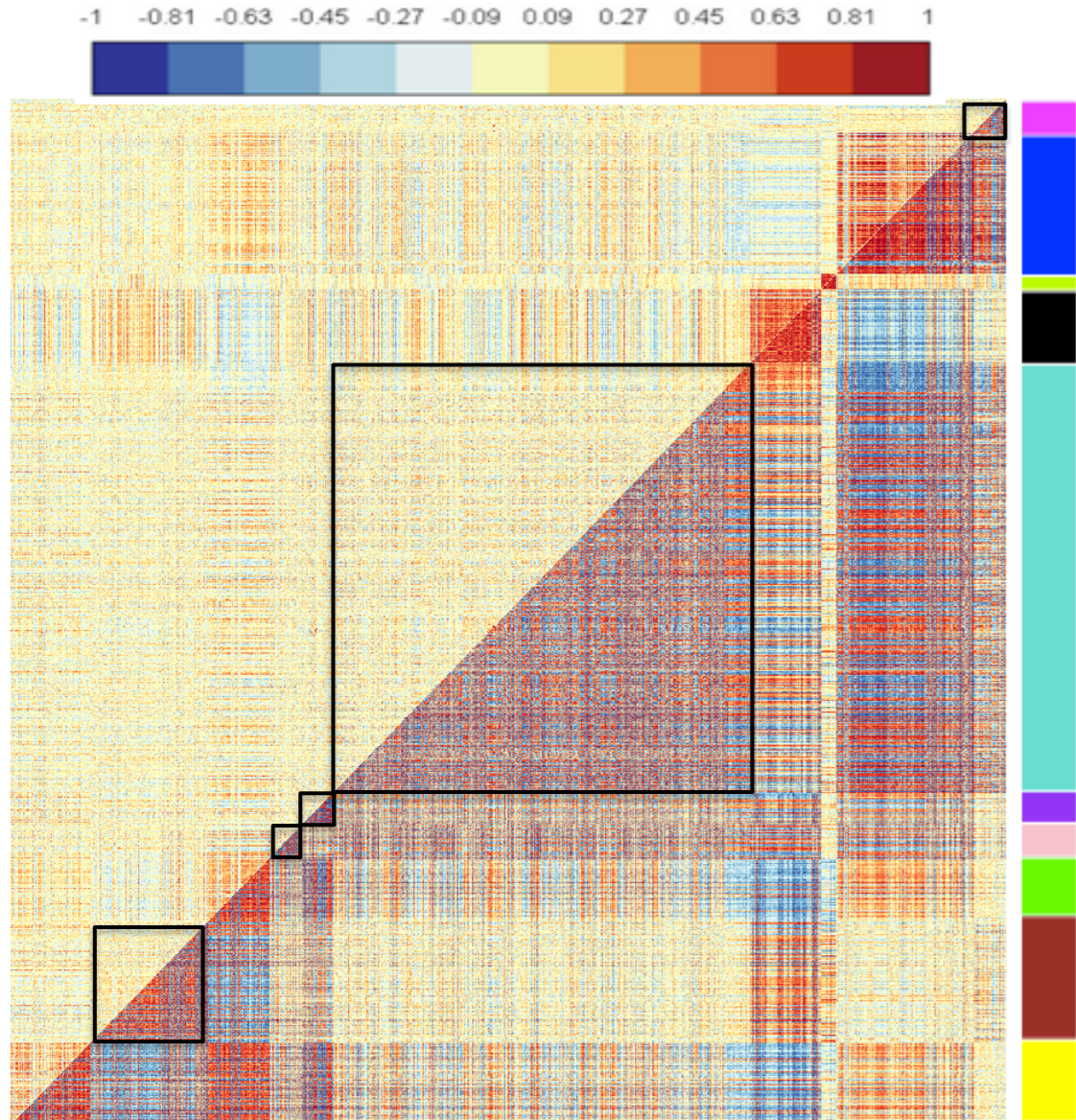

Tumor samples

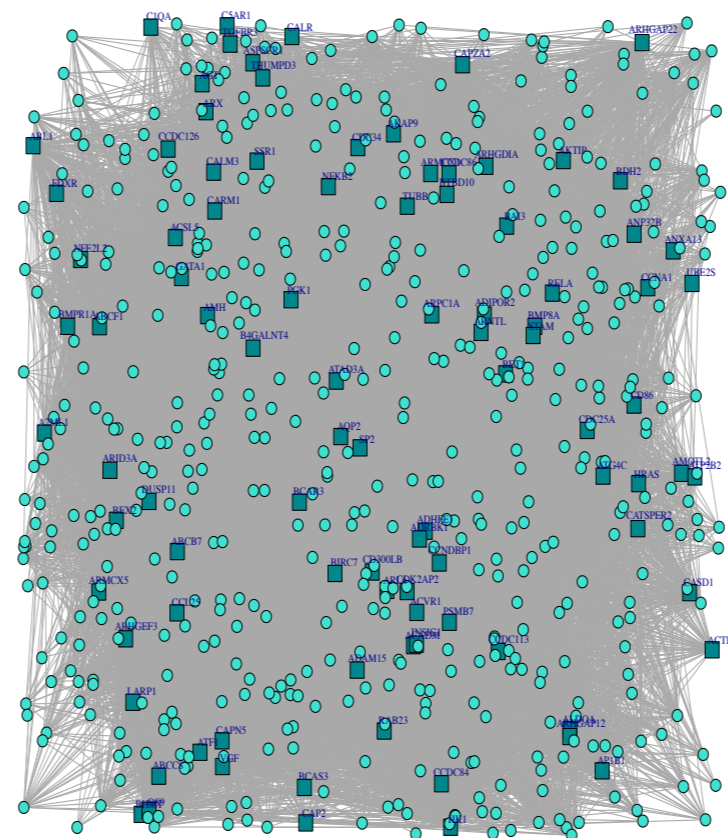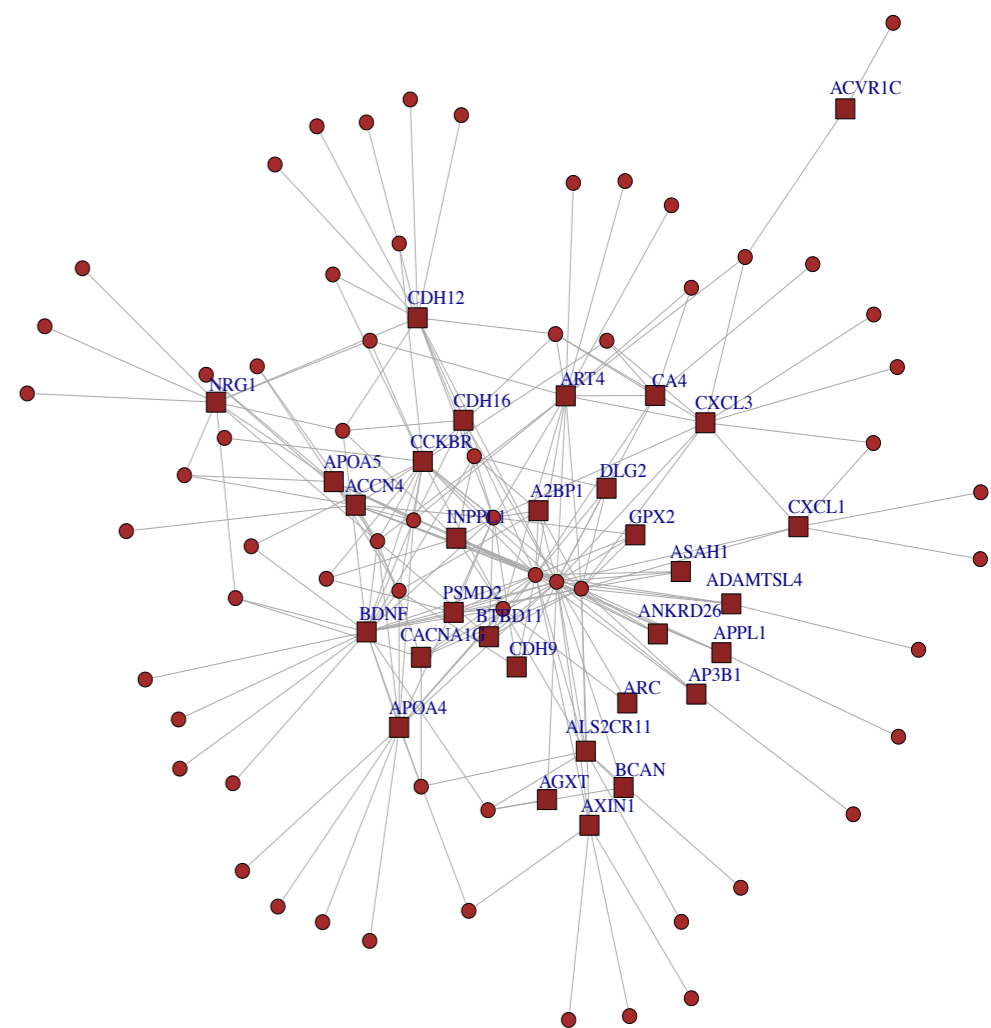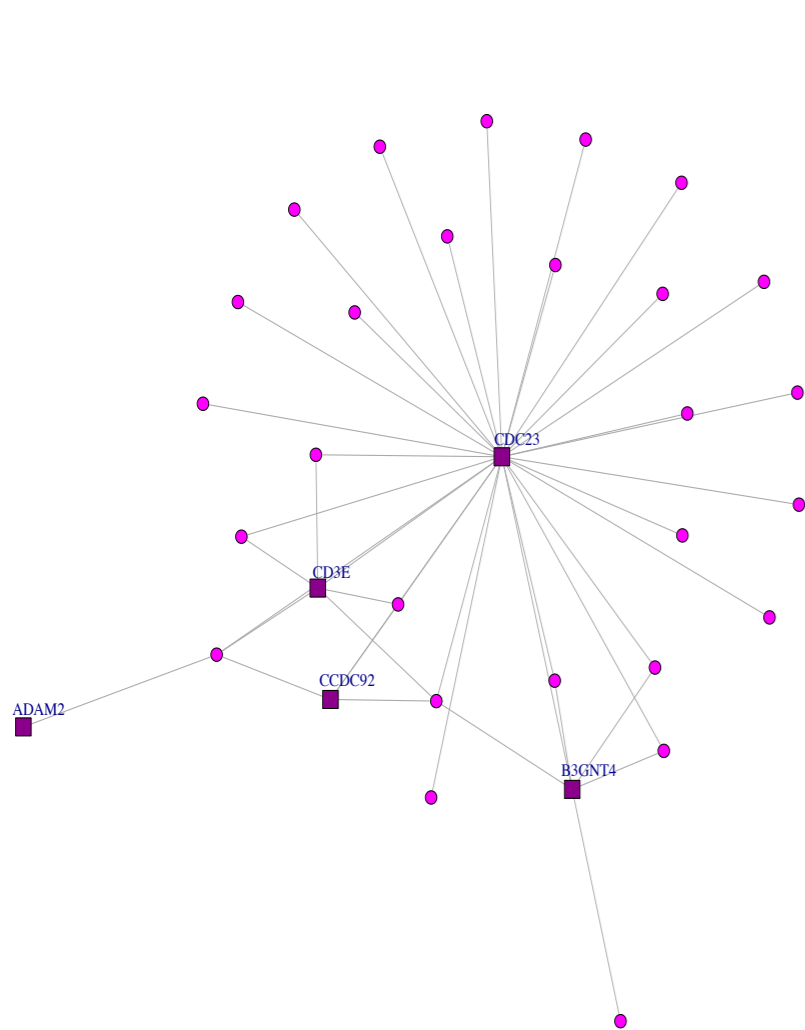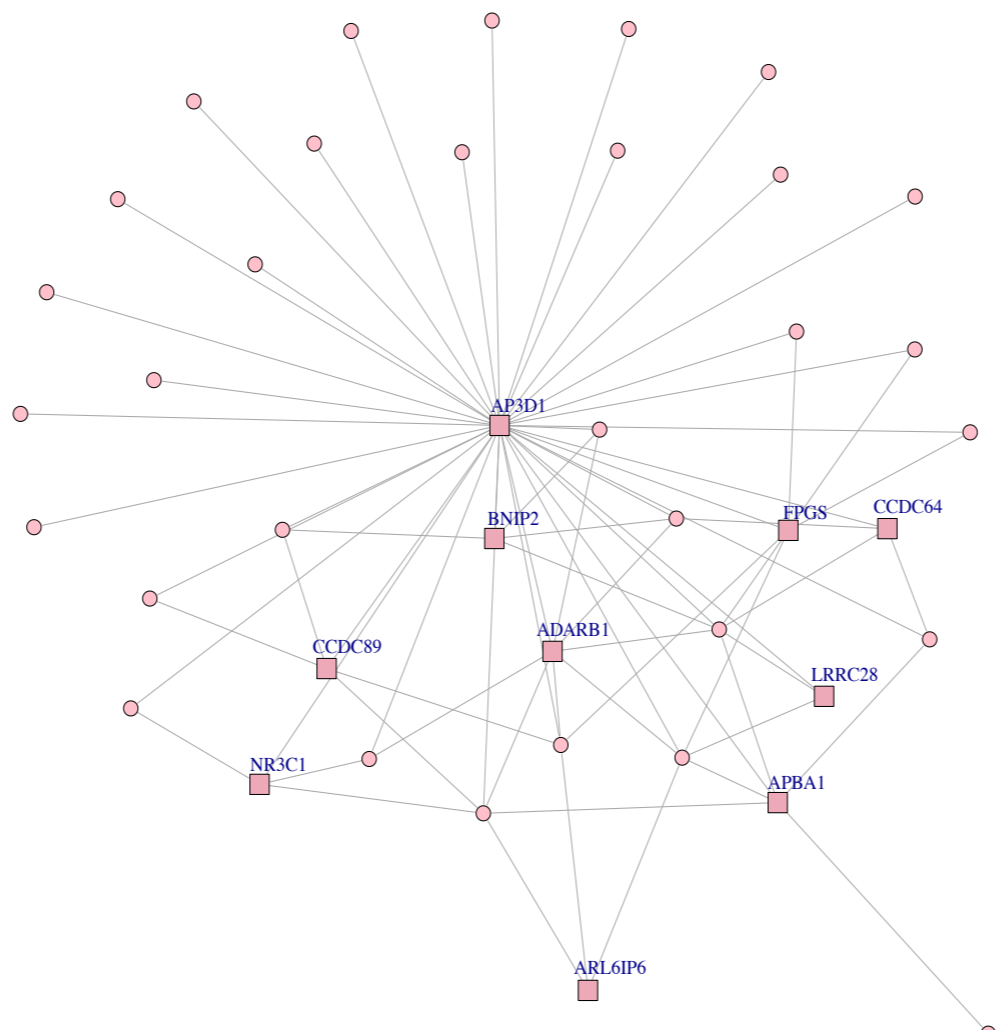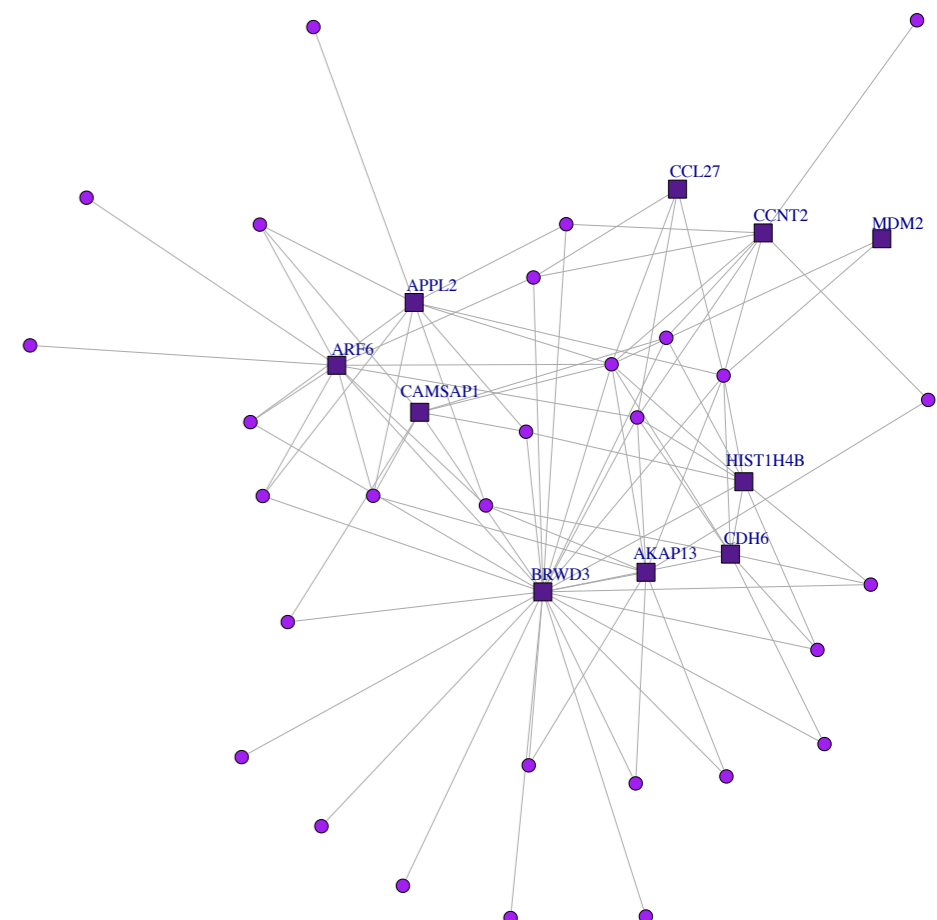

Supplement: Additional file S11 — The network modules inferred using the DiffCoEx method. Each network corresponds to the highlighted module color in the heatmap. For clarity, we visualized only the known drug target genes (labelled and square nodes) and the genes connected to them. [file 1471-2164-16-S5-S2-S11.pdf]
