# Supplementary material for: Integrative network-based approach identifies key genetic elements in breast invasive carcinoma
Source: BMC Genomics. 2015 May 26;16(Suppl 5):S2. doi: 10.1186/1471-2164-16-S5-S2 (PMC4460623; doi:10.1186/1471-2164-16-S5-S2)
Supplement: Additional file S6 — The deregulated miRNAs in proximity to somatic mutations. 21 cases of miRNA-SNV pairs were identified. The genomic distance between miRNAs and SNVs is reported in base pairs. SNVs marked with (*) are the exclusive ones associated only with the dysregulated miRNAs and not with any of the non-dysregulated miRNAs. [file 1471-2164-16-S5-S2-S6.pdf]

miRNA	Chrom	SNP Position	SNP occurring gene	Genomic distance (in bp)
hsa-mir-181b-1	1	198711494 *	PTPRC	116508
hsa-mir-181a-1	1	198711494 *	PTPRC	116679
hsa-mir-1290	1	19186120 *	TAS1R2	37445
hsa-mir-9-1	1	156498803 *	IQGAP3	-108670
hsa-mir-205	1	209605636 *	MIR205HG	-158
hsa-mir-3129	2	189928732	COL5A2	69030
hsa-mir-145	5	148730786 *	GRPEL2	79423
hsa-mir-143	5	148730786 *	GRPEL2	77695
hsa-mir-106b	7	99662436 *	ZNF3	29180
hsa-mir-93	7	99662436 *	ZNF3	28955
hsa-mir-25	7	99662436 *	ZNF3	28747
hsa-mir-320a	8	22136963 *	PIWIL2	-34488
hsa-mir-199b	9	131048299	SWI5	-41299
hsa-mir-199b	9	131023779	GOLGA2	-16779
hsa-mir-152	17	46136186	NFE2L1	-21659
hsa-mir-520d	19	54254529	MIR522	-31179
hsa-mir-519e	19	54254529	MIR522	-71335
hsa-mir-1323	19	54254529	MIR522	-79307
hsa-mir-199a-1	19	10870471	DNM2	57631
hsa-let-7f-2	X	53644041	HUWE1	-59888
hsa-mir-718	X	153278098	IRAK1	7273
