# Supplementary material for: Integrative network-based approach identifies key genetic elements in breast invasive carcinoma
Source: BMC Genomics. 2015 May 26;16(Suppl 5):S2. doi: 10.1186/1471-2164-16-S5-S2 (PMC4460623; doi:10.1186/1471-2164-16-S5-S2)
Supplement: Additional file S8 — A list of the 33 genes whose gene products are targeted by anti-cancer drugs, characterized from the three considered drug databases, CTD, PharmGKB, and Cancer resource. (1) means that at least one drug that targets this gene product is reported in this database, and (0) means no drugs are reported for the respective gene in this database. Not included are substances that are known to be cancerogenous or mutagenic. [file 1471-2164-16-S5-S2-S8.pdf]

Target gene	Drug and antineoplastic agents	CTD	PharmGKB	Cancer Resource
ABCB8	docetaxel; Cyclosporine; Progesterone	1	0	0
ABCG4	indole-3 carbinol; Methotrexate; exemestane; Vincristine	1	0	0
AHCTF1	Methotrexate; bisphenol A	1	0	0
AKT1	U 0126;tyrphostin AG 1478; Ursodeoxycholic Acid;Valproic Acid;tyrphostin AG 1024; trametinib; Tretinoin	1	0	1
APOC1	tanshinone; Quercetin; Fluorouracil; bexarotene; Cisplatin; Tamoxifen	1	0	1
AR	Dihydrotestosterone; Acetylcysteine; celecoxib	1	0	0
ATF6	Nelfinavir; Tretinoin;bisphenol A; Cyclosporine; Curcumin	1	0	0
ATG4C	epigallocatechin gallate	1	0	0
ATP1B1	resveratrol; Ranitidine; vorinostat; Genistein; Progesterone; epigallocatechin gallate	1	0	0
B4GALT7	Cytarabine; Cyclosporine	1	0	0
BIRC6	Dieldrin; Cyclosporine; Cisplatin; Fluorouracil; Doxorubicin; Epirubicin;Estradiol; zoledronic acid; bisphenol A	1	0	0
BRCA1	Tretinoin; trichostatin A; Estradiol; transplatin; troglitazone; Tunicamycin; fulvestrant	1	0	1
CA6	Tretinoin;Carmustine	1	0	0
CCDC130	Quercetin;Tamoxifen;Cyclosporine;bisphenol A	1	0	0
CCDC92	Quercetin; Folic Acid	1	0	0
CD2	Dexamethasone; Methotrexate; Cyclophosphamide	1	0	0
CD79B	Cyclophosphamide	1	0	0
CDC34	Estradiol; bortezomib; Fluorouracil; Tamoxifen	1	0	0
DAPK1	paclitaxel;gemcitabine	0	1	0
EGR1	Fluorouracil; gemcitabine	0	0	1
ESR1	exemestane;tamoxifen	0	1	1
JUN	andrographolide; cinnamic aldehyde; Daunorubicin; decitabine; Cisplatin;Doxorubicin	0	0	1
LRRC28	gemcitabine	0	0	1
MYB	Fluorouracil;gemcitabine;Quercetin	0	0	1
MYC	alitretionoin; Amsarcine; bicalutamide; Camtothecin; decitabine; Cisplatin; Doxorubicin	0	0	1
NFKB1	Curcumin; decitabine; Doorubicin; Echinomycin; Fluorouracil; gefitinib; indole-3-carbinol; parthenolide	0	0	1
NQO2	doxorubicin; cyclophosphamide	0	1	0
OS9	alitretionoin	0	0	1
SP1	Etoposide; indole-3-carbinol; Ionidamine; Quercetin; Adaphostin	0	0	1
STAT3	azaspirane; bisphenol A; Capsaicin; Fluorouracil; interferon alfacon-1; resveratrol;sulindac sulfide; Tamoxifen	0	0	1
TGFB1	Doxorubicin; Fluorouracil; Thalidomide; Entinostat; Hyaluronidase	0	0	1
TP53	4-biphenylmine; alliin; Apigenin; Atropine;bicalutamide;butylidenephthalide	0	0	1
