# Supplementary material for: Integrative network-based approach identifies key genetic elements in breast invasive carcinoma
Source: BMC Genomics. 2015 May 26;16(Suppl 5):S2. doi: 10.1186/1471-2164-16-S5-S2 (PMC4460623; doi:10.1186/1471-2164-16-S5-S2)
Supplement: Additional file S9 — Ten most significant GO terms and KEGG pathways enriched in the list of the 73 candidate driver genes. [file 1471-2164-16-S5-S2-S9.pdf]

Category	Enriched term	P-value
GO functional terms	GO:0006357~regulation of transcription from RNA polymerase II promoter	6.67E-09
	GO:0006355~regulation of transcription, DNA-dependent	1.15E-07
	GO:0006350~transcription	1.59E-07
	GO:0051252~regulation of RNA metabolic process	1.75E-07
	GO:0045449~regulation of transcription	1.96E-07
	GO:0034645~cellular macromolecule biosynthetic process	1.08E-06
	GO:0019219~regulation of nucleobase, nucleoside, nucleotide and nucleic acid metabolic process	1.10E-06
	GO:0010556~regulation of macromolecule biosynthetic process	1.24E-06
	GO:0009059~macromolecule biosynthetic process	1.26E-06
	GO:0051171~regulation of nitrogen compound metabolic process	1.33E-06
KEGG pathways	hsa05223:Non-small cell lung cancer	2.48E-03
	hsa04110:Cell cycle	3.42E-03
	hsa05215:Prostate cancer	1.01E-02
	hsa05219:Bladder cancer	1.91E-02
	hsa05200:Pathways in cancer	2.32E-02
	hsa05214:Glioma	4.06E-02
